# Epigenetic prediction of major depressive disorder

**DOI:** 10.1038/s41380-020-0808-3

**Published:** 2020-06-10

**Authors:** Miruna C. Barbu, Xueyi Shen, Rosie M. Walker, David M. Howard, Kathryn L. Evans, Heather C. Whalley, David J. Porteous, Stewart W. Morris, Ian J. Deary, Yanni Zeng, Riccardo E. Marioni, Toni-Kim Clarke, Andrew M. McIntosh

**Affiliations:** 1 Division of Psychiatry, Centre for Clinical Brain Sciences, University of Edinburgh, Edinburgh, UK; 2 Centre for Genomic and Experimental Medicine, Institute of Genetics and Molecular Medicine, University of Edinburgh, Edinburgh, UK; 3 Centre for Cognitive Ageing and Cognitive Epidemiology, School of Philosophy, Psychology and Language Sciences, University of Edinburgh, Edinburgh, UK; 4 Social Genetic and Developmental Psychiatry Centre, Institute of Psychiatry, Psychology & Neuroscience, King’s College London, London, UK; 5 Faculty of Forensic Medicine, Zhongshan School of Medicine, Sun Yat-Sen University, 74 Zhongshan 2nd Road, Guangzhou, 510080 China; 6 Guangdong Province Translational Forensic Medicine Engineering Technology Research Center Zhongshan School of Medicine, Sun Yat-Sen University, 74 Zhongshan 2nd Road, Guangzhou, China

**Keywords:** Predictive markers, Genetics

## Abstract

Variation in DNA methylation (DNAm) is associated with lifestyle factors such as smoking and body mass index (BMI) but there has been little research exploring its ability to identify individuals with major depressive disorder (MDD). Using penalised regression on genome-wide CpG methylation, we tested whether DNAm risk scores (MRS), trained on 1223 MDD cases and 1824 controls, could discriminate between cases (*n* = 363) and controls (*n* = 1417) in an independent sample, comparing their predictive accuracy to polygenic risk scores (PRS). The MRS explained 1.75% of the variance in MDD (*β* = 0.338, *p* = 1.17 × 10^−7^) and remained associated after adjustment for lifestyle factors (*β* = 0.219, *p* = 0.001, *R*^2^ = 0.68%). When modelled alongside PRS (*β* = 0.384, *p* = 4.69 × 10^−9^) the MRS remained associated with MDD (*β* = 0.327, *p* = 5.66 × 10^−7^). The MRS was also associated with incident cases of MDD who were well at recruitment but went on to develop MDD at a later assessment (*β* = 0.193, *p* = 0.016, *R*^2^ = 0.52%). Heritability analyses found additive genetic effects explained 22% of variance in the MRS, with a further 19% explained by pedigree-associated genetic effects and 16% by the shared couple environment. Smoking status was also strongly associated with MRS (*β* = 0.440, *p* ≤ 2 × 10^−16^). After removing smokers from the training set, the MRS strongly associated with BMI (*β* = 0.053, *p* = 0.021). We tested the association of MRS with 61 behavioural phenotypes and found that whilst PRS were associated with psychosocial and mental health phenotypes, MRS were more strongly associated with lifestyle and sociodemographic factors. DNAm-based risk scores of MDD significantly discriminated MDD cases from controls in an independent dataset and may represent an archive of exposures to lifestyle factors that are relevant to the prediction of MDD.

## Introduction

Major depressive disorder (MDD) is a disabling condition with an estimated point prevalence of 4.4% [1]. Recent genome-wide association studies (GWASs) have begun to elucidate the genetic architecture of MDD [2, 3] and polygenic risk scores (PRS) derived from the most recent study of 246,363 depression cases and 561,190 controls explain 1.5–3.2% of MDD risk in independent cohorts [4]. As sole predictors of MDD status, PRS currently have limited clinical utility and may not capture the larger environmental contributions to risk.

Variation in DNA methylation (DNAm) is affected by both genetic and environmental factors, which act in combination to confer risk for diseases and complex traits [5]. DNAm has recently been studied in relation to MDD [6, 7]. An epigenome-wide association study (EWAS) of 7948 European individuals identified 3 CpG sites that were differentially methylated in association with depressive symptoms [6]. Annotation of these sites implicated genes involved in axon guidance. A study of 150 monozygotic twin pairs discordant for early onset MDD identified 760 differentially methylated CpG sites, which mapped to neuronal circuitry and plasticity genes [7]. These findings suggest that differences in DNAm may be associated with MDD.

Many lifestyle factors associated with MDD, including smoking [8], obesity [9, 10] and alcohol consumption [11], are associated with differential genome-wide DNAm. These DNAm signatures have been leveraged, using penalised regression to identify a subset of informative CpG sites, to create DNAm risk scores (MRS), which can predict the trait of interest in an independent cohort. McCartney et al. showed that DNAm scores explained 61% of the variance in smoking status and 12.5% of the variance in body mass index (BMI) and alcohol consumption. When modelled alongside PRS, DNAm scores contribute additively to the variance explained for these traits [12]. DNAm therefore acts as an archive of exposure to several risk factors for poor mental health, however the significance of its association with MDD remains unexplored.

A recent study of 581 individuals with depressive symptoms used machine learning methods to train a predictor of MDD using DNAm data. They found that MRS could discriminate future MDD disease status with an area under the curve (AUC) of 0.72 [13]. Notably, this study did not use an independent sample to test their MRS and they discriminated between transient and chronic MDD over a 6-year period. The aim of the current study was to use penalised regression to train a predictor of MDD based on DNAm in a large sample using the Generation Scotland: Scottish Family Health Study (GS:SFHS) cohort [14, 15]. A training set of 1223 MDD cases and 1824 controls was used to create an MDD MRS which was then tested in 1970 independent individuals (363 prevalent and 190 incident MDD cases; 1417 controls). As smoking has been consistently associated with differential DNAm [8, 12], we created an MDD MRS that excluded smoking signals (MRS-ns) by training the predictor on a subset of individuals who had never smoked (534 MDD cases and 1017 controls). Using longitudinal clinical data, we also tested whether MRS and MRS-ns derived from blood taken at the baseline assessment would predict future (incident) MDD status at follow-up between 4 and 10 years later. To explore whether the MDD MRS and MRS-ns capture exposure to lifestyle factors associated with MDD, we also tested the association between MDD MRS and MRS-ns, and alcohol use, BMI, smoking status, and pack years, as well as self-reported antidepressant use.

To determine whether the MRS was capturing genetic or environmental liability to MDD, we performed variance component analysis to estimate the single nucleotide polymorphism (SNP)-based and environmental contributions to MDD-associated methylation signatures. Finally, to explore whether the MDD MRS and MRS-ns capture exposure to lifestyle factors associated with MDD, we tested the association between MDD MRS and MRS-ns, and 61 behavioural phenotypes and lifestyle factors. We compared these associations with those observed for PRS that have previously shown association with a wide range of neuropsychiatric traits [16].

## Methods

### Study population

#### Generation Scotland—the Scottish Family Health Study (GS:SFHS)

GS:SFHS is a family-based population cohort investigating the genetics of health and disease in ~24,000 individuals across Scotland [14, 15]. Baseline data were collected between 2006 and 2011. The present study focuses on 5017 individuals for whom DNAm data from a blood draw at baseline contact, baseline phenotypic data and genotype data were available. Environmental data, such as lifestyle factors, were also measured (BMI) or recorded (smoking status and alcohol consumption) on nearly all study participants.

Longitudinal phenotypic data are available for a subset of individuals who responded to a recontact request [17, 18]. For these individuals we have information on MDD case-control status both at baseline and follow-up, which occurred 4–10 years later (2015–2016). GS:SFHS received ethical approval from NHS Tayside Research Ethics Committee (REC reference number 05/S1401/89) and has Research Tissue Bank Status (reference: 15/ES/0040). Written informed consent was obtained from all participants.

### Phenotypes

BMI was calculated using height (cm) and weight (kg) measured by clinical staff during baseline recruitment. Alcohol intake was self-reported as part of a pre-clinical questionnaire. Participants were asked whether they were “never”, “former” or “current” drinkers. Current drinkers were asked: “During the past week, please record how many units of alcohol you have had”.

Smoking status was recorded by asking participants: “Have you ever smoked tobacco?”. Answers were recorded as: “Yes, currently smoke; Yes, but stopped within the past 12 months; Yes, but stopped more than 12 months ago; No, never smoked”. Previous evidence has shown that cigarette smoking has strong associations with genome-wide DNAm, and effects persist long after smoking cessation, indicating that former smokers may retain DNAm profiles that are similar to current smokers [10, 19]. Therefore, for the current study, we assigned smoking status as a binary variable, by converting all “Yes” answers to smoker [1], and “No” to non-smoker (0). Using smoking behaviour data, pack years were calculated by multiplying the number of cigarette packs (20 cigarettes/pack) smoked per day by the number of years a person has smoked [20].

Antidepressant use was self-reported by participants at the baseline assessment and has been described in greater detail previously ([21]; Supplementary material). See Supplementary Tables 1 and 2 also for demographic differences in lifestyle factors between individuals with an MDD diagnosis and those without one.

Baseline MDD status was measured using the axis-I Structured Clinical Interview of the Diagnostic and Statistical Manual, version IV (SCID) and was administered to participants who answered “yes” to either of two screening questions (see Supplementary materials). MDD status was measured prospectively by remote paper questionnaire between 4 and 10 years after baseline assessment (2015–2016) using the Composite International Diagnostic Interview—Short Form (CIDI-SF) as described previously [17].

Control participants were defined as those individuals who answered “no” to the two screening questions (see Supplementary materials) and did not fulfil criteria for a diagnosis of current or previous MDD following the SCID interview and CIDI-SF remote follow-up assessment. Individuals fulfilling criteria for schizophrenia or bipolar disorder, or who self-reported these diagnoses, were also excluded from both case and control groups.

### DNA methylation

In total, 9873 individuals in GS:SFHS had genome-wide DNAm data profiled from blood samples using the Illumina Human-MethylationEPIC BeadChip. The raw data were acquired, preprocessed and quality checked in two different batches, hereafter named batch 1 (*n* = 5190) and batch 2 (*n* = 4588).

In batch 1, ShinyMethyl [22] was used to exclude samples where predicted sex mismatched recorded sex, as well as to plot the log median intensity of methylated and unmethylated signals per array and inspect the output from the control probes; outlying samples detected by visual inspection were excluded. WateRmelon [23] was then used to remove probes in which >1% of probes had a detection *p* value > 0.05; probes with a beadcount of <3 in more than 5% samples; and probes in which >5% of samples had a detection *p* value > 0.05 [12]. Multi-dimensional scaling (MDS) plots were inspected to confirm that there were no additional sample outliers. WateRmelon was then used to normalise the data using the dasen method, and lumi [24] was used for conversion to *M* values, which were then pre-corrected for relatedness, estimated blood cell types, and processing batch using DISSECT [25], for CpG sites on autosomal chromosomes. The final dataset comprised corrected *M* values at 841,753 loci measured for 5087 individuals.

In batch 2, Meffil [26] and ShinyMethyl [22] were used for quality control of the raw data. Using Meffil, samples were removed if: there was a mismatch between self-reported and methylation-predicted sex; they had >1% of CpG sites with a detection *p* value > 0.05; they showed evidence of dye bias; they were outliers for the bisulphite conversion control probes; and had a median methylated signal intensity > 3 standard deviations lower than expected. Afterwards, ShinyMethyl was used to perform further quality control, as described above for batch 1. MDS plots were inspected, and outliers were excluded. Meffil was then used again to identify and exclude poor-performing probes, which were deemed as such if: they had a beadcount of <3 in >5% samples and/or >5% samples had a detection *p* value > 0.05. The data were normalised using the dasen method in wateRmelon, and the beta2m function in lumi [24] was used to generate *M* values. The final dataset comprised *M* values for 773,860 loci measured in 4450 individuals.

### Genotyping and PRS profiling

Individuals were genotyped using the Illumina OmniExpress BeadChip. The raw genotype data underwent a series of quality control steps: individuals with a call rate < 98%, SNPs with a genotype rate < 98%, minor allele frequency < 1%, and Hardy–Weinberg *p* value < 10^−6^ were removed from the initial dataset and then imputation was performed using the Sanger Imputation Service with the Haplotype Reference Consortium panel v1.1 [27].

Using the largest available depression GWAS [4], depression PRS were computed using Plink v1.90b4 [28] using SNPs that met a significance level of *p* ≤ 0.05, in line with previous studies, which have shown that this threshold explains the most variance in MDD status [4]. GWAS summary statistics excluding GS:SFHS were obtained in order to create PRS in the GS:SFHS sample. Clumping was applied using a linkage disequilibrium *r*^2^ < 0.1 and a 500-kb window.

### DNAm predictor—training and testing datasets

In order to obtain a training and testing dataset, individuals were separated based on the two batches described above. Supplementary Fig. 1 provides a flowchart summary of the analysis process.

#### Training dataset

Batch 1 was used to train two DNAm predictors. The dataset consisted of controls who were screened as unaffected (*n* = 1824) at both baseline and follow-up (i.e., answered “no” to screening questions at baseline and follow-up), or who screened positive but were subsequently found not to fulfil diagnostic criteria for MDD using the SCID. MDD cases were those who screened positive for depression by answering yes to one or more brief screening questions and who subsequently fulfilled criteria for MDD at baseline SCID interview (*n* = 1223). The non-smoker dataset was created by excluding those individuals who had a smoking history from the entire batch 1 dataset (*N* excluded = 1496; i.e. answered “yes” to the question “have you ever smoked tobacco?”). As such, this dataset contained 1017 controls who were screened as unaffected at both baseline and follow-up and 534 MDD cases who were screened positive for depression who answered “no, never smoked” to the question “have you ever smoked tobacco?”.

CpG sites measured in the individuals mentioned above were included as independent variables in a least absolute shrinkage and selection operator (LASSO) penalised regression model described below. Depression status was regressed on age, sex and ten genetic principal components, and the extracted residuals from this model were input as the dependent variable in the LASSO regression model.

LASSO penalised regression models were run using the “glmnet” function in R in order to train DNAm predictors. We applied tenfold cross-validation and the mixing parameter was set to 1 for our LASSO penalty.

#### Testing dataset

Batch 2 was used in order to create MDD MRS using the CpG sites identified in the training set using LASSO regression models. Using the set of CpG sites selected from the penalised regression, MRS were calculated in the testing dataset using the weights estimated in the training set, first for prevalent depression (Total *n* = 1780; cases = 363; controls = 1417) and then for incident depression (Total *n* = 1607; cases = 190; controls = 1417). Prevalent depression refers to those individuals who were depressed at both baseline and follow-up, while incident depression refers to those individuals who were well at baseline but went on to develop MDD.

### Statistical methods

All analyses were conducted using R (version 3.2.3) in a Linux environment. The R code for the current analyses is available in Supplementary materials.

#### Association of MRS and MRS-ns with depression

The association between both MDD MRS and MDD status was assessed using logistic regression. We tested the association between MRS and prevalent depression (Total *n* = 1780; cases = 363; controls = 1417) and between MRS and incident depression (Total *n* = 1607; cases = 190; controls = 1417). We repeated these analyses using the MRS-ns score and also performed sensitivity analyses by selecting individuals who had self-reported antidepressant use (Total *N*_Prevalent_ = 1250, cases = 198, controls = 1052; Total *N*_Incident_ = 1195, cases = 143, controls = 1052). McFadden’s *R*^2^ were calculated to determine the amount of variance in MDD explained by MRS.

We tested whether lifestyle factors previously shown to be associated with both MDD and DNAm [8–12] were associated with the MRS. Using linear regression, we tested whether MRS and MRS-ns were associated with BMI, pack years and alcohol consumption. Logistic regression models were used to test whether MRS and MRS-ns were associated with self-reported antidepressant use and smoking status. To estimate how much variance MRS and MRS-ns explain in MDD status when adjusting for lifestyle factors, MDD status was modelled as a dependent variable with alcohol consumption, BMI, smoking and pack years fit as covariates. We also tested the effect of fitting self-reported antidepressant use in our models to determine whether the MRS and MRS-ns would still significantly contribute to the risk for MDD. This was carried out for both incident and prevalent cases.

In addition, using the “ROCR” R package, we plotted the predictive ability of MRS and MRS-ns in both incident and prevalent cases and controls using a Receiver Operating Characteristic (ROC) curve, representing the sensitivity and specificity of the score in relation to depression.

#### Mediation analysis

Mediation analysis was carried out to illustrate the relationship between PRS, MRS and MDD. In two separate mediation models, PRS was set as the independent variable, MRS or MRS-ns as the mediator, and MDD as the outcome. All variables were adjusted for age and sex. In addition, PRS and MRS/MRS-ns were adjusted for the first ten genetic principal components, and MRS/MRS-ns were adjusted for BMI, alcohol consumption and smoking status.

In addition, two mediation models were tested in order to illustrate the relationship between the MRS, smoking and MDD. In the first model, MDD was set as the independent variable, smoking as the mediator and the MRS as the outcome. MDD and smoking were adjusted for age and sex. MRS was adjusted for age, sex, batch and the first ten genetic principal components. In the second model, smoking was set as the independent variable, MRS as the mediator and MDD as the outcome. Covariates for the three variables were consistent with the first mediation model.

The ‘lavvan’ package in R was used for mediation analyses [29]. Categorical variables such as sex were transformed into numeric. All variables including covariates were scaled to a mean of 0 and a standard deviation of 1.

#### Heritability and environmental contributions to MRS and identification of mQTLs

A recent study using 5101 individuals from the present GS:SFHS cohort determined the relative genetic and environmental contributions to DNAm at each CpG site on the EPIC illumina array [5]. This study used a five-component variance component method [30], which estimates the SNP (*G*) and additional genetic (kinship) (*K*) contributions to methylation variance alongside contributions from the nuclear family (*F*), couple (*C*) and sibling (*S*) shared environments. Using this approach, we tested the genetic and environmental contributions to the total MRS/MRS-ns using the GKFCS method [30]. Briefly, this decomposes variation in the MRS into the two genetic components (*G* and *K*) and three environmental components (C, F and S) using GCTA software [30]. Using a backward-stepwise selection model, we initially fit all five components and then dropped components if they were not significant using Wald or likelihood ratio tests (LRT). This process was repeated until all components significantly contributed to variance in the MRS. This method and the construction of the GKFCS matrices has been previously described in more detail in Xia et al. (2016) [30] and Zeng et al. [5]. We also assessed whether SNPs associated with methylation (mQTLs) at the CpG sites which comprise the MRS were enriched for association with MDD. mQTLs for MRS CpG sites were identified using the ARIES dataset using the midlife timepoint (http://www.mqtldb.org/) [31]. Significant mQTLs were then tested for their association with MDD by performing a look-up of their *p* values from summary statistics of the largest GWAS of MDD to date [4]. False discovery rate (FDR) correction was then performed on the MDD *p* values to identify those mQTLs significantly associated with both CpG methylation and MDD status after correction for multiple testing.

#### MRS and PRS associations with an archive of 61 behavioural variables

The GLM function in R was used to test associations between the MRS and MDD PRS, which were set as predictor variables, and 61 behavioural, cognitive and lifestyle phenotypes, which were included as outcome variables (for a list of all phenotypes included in the analysis, please see Supplementary Table 3). Covariates in all models included age, sex and ten genetic principal components. FDR correction was applied over all tests (61 outcome variables × MRS + PRS) using the p.adjust function in R (*q* < 0.05).

#### Pathway analysis

To annotate CpG sites comprising the MRS, we used the Infinium MethylationEPIC BeadChip database, which provides information concerning genes, chromosome location, start and end site and other characteristics (https://emea.support.illumina.com/array/array_kits/infinium-methylationepic-beadchip-kit/downloads.html). We then used the Functional Mapping and Annotation of Genome-wide association studies (FUMA; 32) in order to identify biological pathways that are associated with the annotated genes (see Supplementary Excel Files 1A and 1B for a list of CpG sites and annotated genes for MRS and MRS-ns, respectively). The database tests whether genes of interest are overrepresented in any pre-defined gene sets across a number of databases. We interrogated a number of gene sets using data from the Gene Ontology (GO) Consortium using the FUMA online tool [32].

## Results

One hundred and ninety-six CpG sites were selected for the MRS predictor that corresponded to the minimum mean cross-validated error within the entire training dataset (*n* = 3047). Similarly, 144 CpG sites were included in the non-smoker training dataset (*n* = 1,551) (see Supplementary Excel Files 2A and 2B for a list of CpG sites and their regression weights for the MRS and MRS-ns, respectively).

### Association of MRS with depression

The MRS was significantly associated with both prevalent (Total *n* = 1780; cases = 363, controls = 1417; *β* = 0.338, *p* = 1.17 × 10^−7^) and incident (Total *n* = 1607; cases = 190, controls = 1417; *β* = 0.193, *p* = 0.016) MDD in the testing dataset; however the MRS explained 1.75% of the variance of prevalent MDD and only 0.52% of incident MDD. The MRS trained on non-smokers (MRS-ns) was significantly associated with prevalent MDD (Total *n* = 1780; cases = 363, controls = 1417; *β* = 0.157, *p* = 0.015), but only explained 0.4% of the variance in MDD status. The MRS-ns was not significantly associated with incident MDD (Total *n* = 1607; cases = 190, controls = 1417; *β* = 0.109, *p* = 0.173, *R*^2^ = 0.196%).

**Fig. 1:**
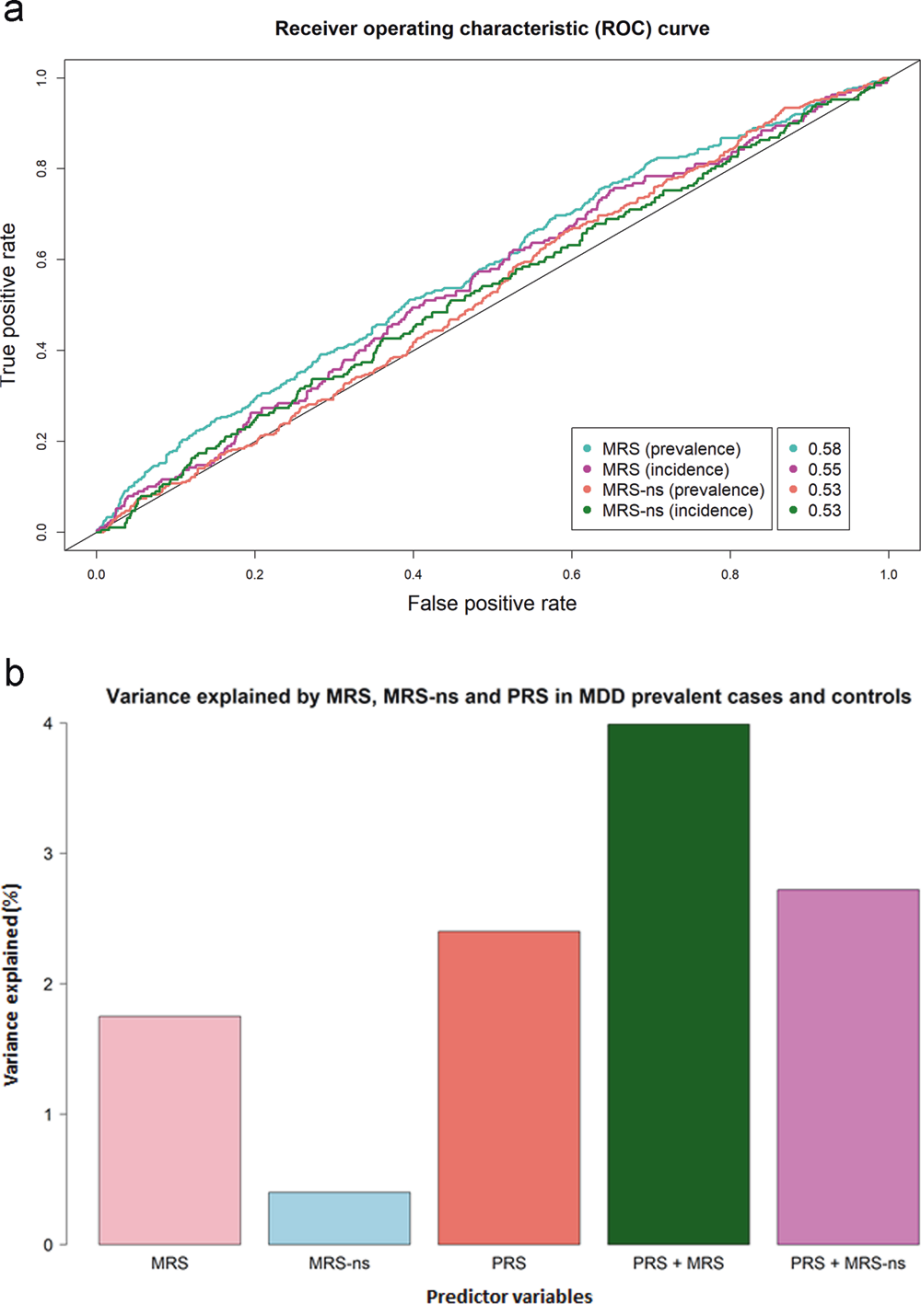
Prediction of MDD case-control status. **a** Receiver Operating Characteristic (ROC) curve indicating the sensitivity (*y*-axis) and specificity (*x*-axis) of methylation risk score (MRS) and methylation risk score trained on non-smokers (MRS-ns) for both prevalent and incident MDD. The AUC estimates are indicated for each predictor in the legend. **b** Variance in prevalent MDD (indicated by *R*^2^ (%) on the y-axis) explained by MRS and PRS alone when fitting MDD as the outcome variable and fitting age, sex and ten genetics principal components as covariates. MRS and PRS are then fit in the same model (PRS + MRS) to show their additive contribution to variance explained in MDD.

After adjustment for self-reported antidepressant use, MRS was still significantly associated with prevalent MDD (*β* = 0.236, *p* = 0.004, *R*^2^ = 0.77%), as was MRS-ns (*β* = 0.191, *p* = 0.015, *R*^2^ = 0.54%). The association with incident MDD after adjusting for antidepressant use was positive but not significant (*β* = 0.091, *p* = 0.296, *R*^2^ = 0.121%). See Fig. 1a for a ROC curve showing the ability of MRS and MRS-ns to discriminate between MDD cases and controls; for MRS, the AUC was 0.58 in prevalent and 0.55 in incident depression, while for MRS-ns, the AUC was 0.53 for both prevalent and incident depression.

Both MRS (*β* = 0.338, *p* = 1.17 × 10^−7^, *R*^2^ = 1.75%) and PRS (*β* = 0.397, *p* = 1.02 × 10^−9^, *R*^2^ = 2.40%) accounted for a small proportion of the variance in risk of prevalent MDD. The model including both MRS (*β* = 0.327, *p* = 5.66 × 10^−7^) and PRS (*β* = 0.384, *p* = 4.69 × 10^−9^) demonstrated that these two risk scores act additively (*R*^2^ = 3.99%) and we found no evidence of an interaction (*β* = −0.009, *p* = 0.892) (Supplementary Table 4). The model including both MRS-ns (*β* = 0.142, *p* = 0.032) and PRS (*β* = 0.394, *p* = 1.39 × 10^−9^) also found an additive effect of both scores (*R*^2^ = 2.72%) with no evidence of an interaction (*β* = 0.049, *p* = 0.483). Figure 1b shows the variance in MDD explained (%) by MRS, MRS-ns and PRS.

We performed sensitivity analyses using MDD cases and controls with no self-reported antidepressant use (Total *N*_Prevalent_ = 1250, cases = 198, controls = 1052; Total *N*_Incident_ = 1195, cases = 143, controls = 1052), MRS was significantly associated with prevalent (*β* = 0.331, *p* = 6.19 × 10^−5^, *R*^2^ = 1.66%) and incident (*β* = 0.232, *p* = 0.011, *R*^2^ = 0.76%) MDD. The variance explained in the antidepressant-free subset was slightly lower compared with the full prevalent case-control sample (antidepressant-free sample: *R*^2^ = 1.66%; full sample: *R*^2^ = 1.75%). MRS-ns was significantly associated with prevalent MDD with no antidepressant use (*β* = 0.189, *p* = 0.026, *R*^2^ = 0.507%).

### Association of MRS and MRS-ns with lifestyle factors and self-reported antidepressant use

The MRS was associated with smoking status (*β* = 0.440, *p* ≤ 2 × 10^−16^, *R*^2^ = 3.2%), pack years (*β* = 0.246, *p* ≤ 2 × 10^−16^, *R*^2^ = 6.5%), alcohol consumption (*β* = 0.092, *p* = 9.85 × 10^−5^, *R*^2^ = 0.7%) and self-reported antidepressant use (*β* = 0.289, *p* = 0.002, *R*^2^ = 1.1%). When partitioning smokers into current and former smokers, the MRS was associated with both (current smokers vs controls: *β* = 1.096, *p* ≤ 2 × 10^−16^, *R*^2^ = 15.53%; former smokers vs controls: *β* = 0.262, *p* = 4.41 × 10^−6^, *R*^2^ = 1.06%). BMI was not significantly associated with MRS (*β* = 0.039, *p* = 0.099, *R*^2^ = 0.097%) (Supplementary Table 5; Supplementary Fig. 2).

The MRS-ns (trained on non-smokers) was also associated with smoking status (*β* = 0.102, *p* = 0.035, *R*^2^ = 0.22%) and pack years (*β* = 0.055, *p* = 0.014, *R*^2^ = 0.27%) in an independent dataset, but the strength of association was attenuated compared with the original MRS. The MRS-ns was still associated with smoking status using current smokers only as cases (*β* = 0.256, *p* = 0.002, *R*^2^ = 0.97%). MRS-ns was not associated with former smoking when these individuals were compared with controls (*β* = 0.059, *p* = 0.264, *R*^2^ = 0.092%). MRS-ns showed a stronger association with BMI (*β* = 0.053, *p* = 0.021, *R*^2^ = 0.246%) than the MRS (*β* = 0.039, *p* = 0.099, *R*^2^ = 0.097%). Alcohol consumption (*β* = 0.024, *p* = 0.289, *R*^2^ = 0.01%) and self-reported antidepressant use (*β* = 0.084, *p* = 0.365, *R*^2^ = 0.096%) were not associated with MRS-ns (Supplementary Table 5; Supplementary Fig. 3).

### Association of MRS and MRS-ns with depression when adjusting for lifestyle factors

**Fig. 2:**
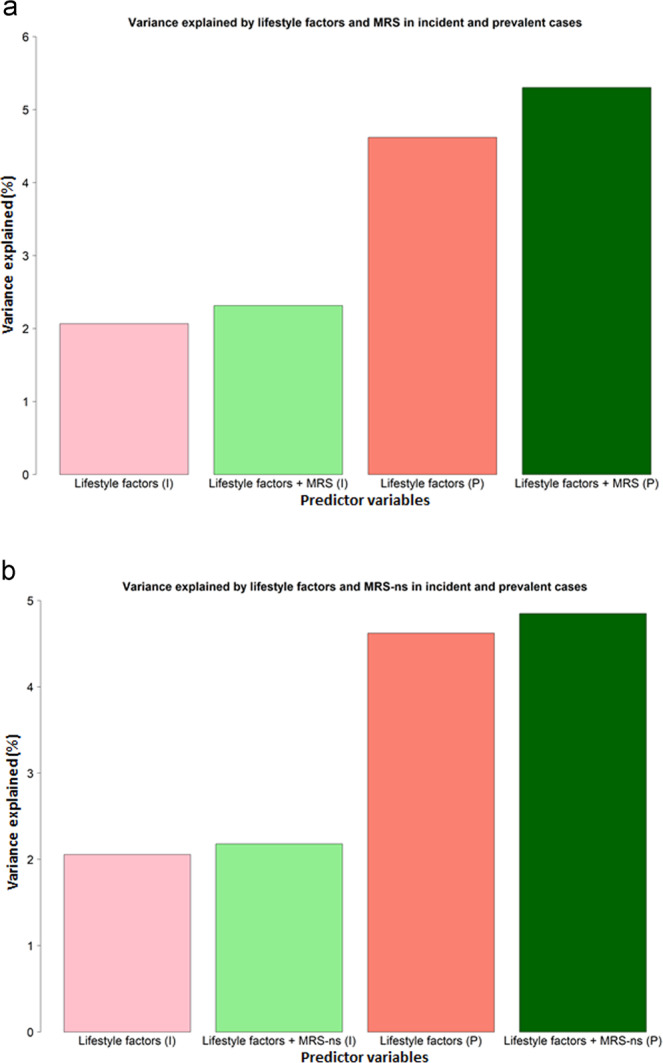
**a** Variance in MDD (indicated by *R*^2^ (%) on the *y*-axis) explained by four lifestyle factors and MRS. **b** Variance in MDD (indicated by *R*^2^ (%) on the *y*-axis) explained by four lifestyle factors and MRS-ns. Lifestyle factors = BMI, alcohol consumption, smoking status and pack years. Light and dark pink bars indicate the additive variance explained by all lifestyle factors combined in incident (I) and prevalent (P) MDD; the light and dark green bars indicate the additive variance explained by all lifestyle factors with the addition of the MRS to the model.

MRS was tested for its association with prevalent and incident depression while adjusting for BMI, alcohol use, smoking status and pack years (lifestyle factors) to determine if any independent contribution remained from the MRS (Table 1 and Fig. 2a). MRS was still associated with prevalent MDD status after adjusting for lifestyle factors (*β* = 0.219, *p* = 0.001) but only explained 0.68% of the variance (compared with *R*^2^ = 1.75% in the unadjusted model). For incident depression cases, the MRS was no longer associated with MDD status after adjusting for lifestyle factors (variance explained decreased from 0.52% prior to adjustment to 0.25% after adjustment).

**Table 1:** Association between four lifestyle factors (BMI, smoking status, pack years, alcohol units), MRS, MRS-ns, and prevalent and incident MDD.

MRS					MRS-ns				
Prevalent MDD	Effect size	SE	*p* value	*R*^2^ (%)	Prevalent MDD	Effect size	SE	*p* value	*R*^2^ (%)
Body mass index	0.256	0.061	2.16 × 10^−5^	1.36	Body mass index	0.256	0.061	2.82 × 10^−5^	1.36
Smoking status	0.369	0.154	0.017	2.13	Smoking status	0.406	0.153	0.008	2.13
Pack years	0.239	0.074	0.001	1.003	Pack years	0.279	0.073	0.0001	1.003
Alcohol units	0.08	0.066	0.232	0.13	Alcohol units	0.092	0.066	0.162	0.13
MRS	0.219	0.067	0.001	0.68	MRS-ns	0.116	0.066	0.08	0.227
Incident MDD	Effect size	SE	*p* value	*R*^2^(%)	Incident MDD	Effect size	SE	*p* value	*R*^2^(%)
Body mass index	0.138	0.076	0.07	0.45	Body Mass Index	0.136	0.076	0.076	0.45
Smoking status	0.629	0.19	0.0009	1.5	Smoking status	0.642	0.189	0.0007	1.49
Pack years	−0.0003	0.099	0.997	0.005	Pack years	0.026	0.098	0.794	0.005
Alcohol units	−0.109	0.095	0.248	0.11	Alcohol units	−0.105	0.094	0.268	0.11
MRS	0.136	0.083	0.1	0.25	MRS-ns	0.085	0.081	0.294	0.124

Results presented are those taken from models fitting all lifestyle factors alongside the two MRS in separate models. Effect sizes represent standardised betas. *R*^2^ represents the variance explained in prevalent and incident MDD by each of the predictor variables.

*SE* standard error, *MRS* methylation risk score, *MRS-ns* methylation risk score trained on non-smokers.

Table 1 and Fig. 2b detail the results for the MRS-ns associations. MRS-ns was not associated with prevalent MDD status after adjusting for lifestyle factors (*β* = 0.116, *p* = 0.08, *R*^2^ = 0.227%).

### Mediation analysis

There was no evidence of mediation or interaction effects of MRS on the relationship between PRS and MDD (Supplementary Figs. 4 and 5).

Smoking significantly mediated the association between MDD and MRS (*β* = 0.071, *p* < 0.001, CFI = 0.976, TLI = 0.954, RMSEA = 0.017), with 52.2% of the mediation taking place through this lifestyle factor (direct association between MDD and MRS before and after adding smoking as the mediator: *C* = 0.136, *C*′ = 0.065).

The MRS mediated the association between smoking and MDD (*β* = 0.019, *p* = 0.008, CFI = 0.977, TLI = 0.995, RMSEA = 0.017). A smaller proportion of variance of 8.51% was mediated by the MRS (direct association between smoking and MDD before and after adding MRS as the mediator: *C* = 0.233, *C*′ = 0.214).

### Heritability and environmental contributions to MRS

Zeng et al. reported that SNP genetic effects (G) explained 9.5% of the variance in CpG methylation across the genome and the additional pedigree effects accounted for 7.2% of the variance [5]. They found little contribution for the shared environment influencing methylation status. We found significant genetic contributions to the MRS total scores (*G* = 0.22 [S.E. = 0.07]), *K* = 0.19 [S.E. = 0.09]), and also significant contributions from the shared couple environment 1(*C* = 0.16 [S.E. = 0.06]), but not from the shared sibling environment (*S* = 1 × 10^−7^ [S.E = 0.03]). A similar pattern was observed for the MRS-ns where genetic effects contributed to a proportion of the observed variance (*G* = 0.22, S.E = 0.07; *K* = 0.19, S.E = 0.08). The recent shared couple environment explained 15% of the variance in the MRS-ns (S.E = 0.06), while the shared sibling environment explained only 6% of the variance in the MRS-ns (S.E. = 0.03).

Methylation quantitative trait loci (mQTLs) were identified for each CpG comprising the MRS using the ARIES dataset mQTLdb [31] using the middle-age timepoint. Seventy-one of the 196 CpG sites had mQTLs (9740 mQTLs in total). We then tested their association with MDD using summary statistics from the largest GWAS of MDD [4]. MDD GWAS data were available for 8327/9740 mQTL. After FDR correction 536 mQTL were significantly associated with MDD and these spanned 11 CpG sites (Supplementary Table 6).

### MRS and PRS associations with an archive of 61 behavioural variables

**Fig. 3:**
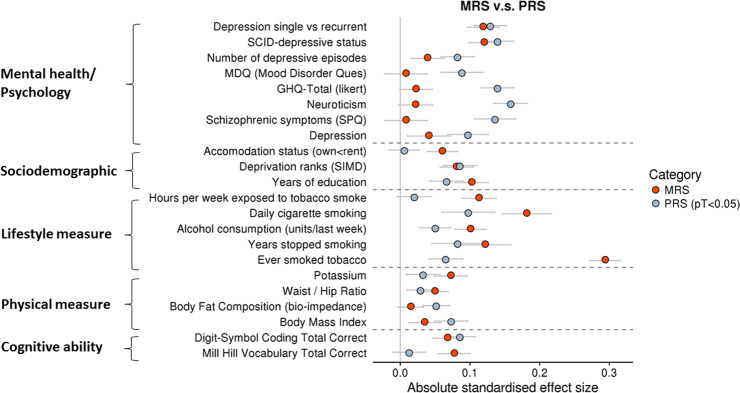
Phenotypic associations with MRS and PRS. Associations between mental health, sociodemographic, lifestyle, physical and cognitive measures and methylation risk score (MRS) in red and polygenic risk score (PRS) in blue; the *x*-axis represents the standardised effect size for each outcome variable listed on the *y*-axis. Error bars represent standard errors of the effect size.

Of 61 variables investigated, we found 8 phenotypes (categories included cognition, lifestyle, physical and sociodemographic measures) associated with the MRS and 10 phenotypes (categories included cognition, lifestyle, sociodemographic, mental health, physical and disease measures) associated with the PRS. Supplementary Table 7 details the results, including standardised effect size, *p* value and p-corrected value for each outcome variable. Figure 3 depicts the absolute effect size for significant outcome variables for MRS compared with PRS. Supplementary Fig. 6 depicts the relationship between MRS and MRS-ns and several outcome variables.

### Pathway analysis

We annotated 159 genes to the 196 CpG sites within the MRS, and 111 genes to the 144 CpG sites within the MRS-ns and used FUMA to identify GO Consortium gene sets enriched for these genes. See Supplementary materials Figs. 7–11 for the GO gene sets, participating genes annotated to the two risk scores, and their respective *p* value for each gene set, for both MRS and MRS-ns.

Sixty-two significant putative gene sets (*P*_corrected_ < 0.05) were identified from the MRS. Of these, 55 were biological processes, of which most included regulation of cellular and molecular processes. Only one of these processes was located in the nervous system: go neurogenesis, involving the generation of cells within the nervous system.

Seventy-two significant putative gene sets (*P*_corrected_ < 0.05) were enriched for the MRS-ns genes. Ten of these gene sets involved biological processes occurring in the brain and nervous system and include neurogenesis, neuron differentiation, neuron projection guidance, dopaminergic neuron differentiation, central nervous system development and forebrain development. Fourteen cellular components were identified, eight of which were located in the nervous system and included the following GO gene sets: neuron part, synapse, neuron projection, axon initial segment, paranode region of axon and node of Ranvier. The full lists of biological and cellular components for the MRS and MRS-ns can be found in Supplementary materials (Figs. 7–11).

Both the biological processes and cellular components identified indicate that the MRS-ns is enriched for annotated genes involved in neurodevelopment across multiple areas within the brain, whereas the genes annotated to the MRS have more broad biological functions not specific to the nervous system.

## Discussion

In the current study, we created a methylation risk score for MDD and investigated its association with prevalent depression (individuals who were depressed at both baseline and follow-up) and whether altered DNAm at baseline predicted incident depression between 4 and 10 years later. Our MRS explained 1.75% of the variance in prevalent MDD compared with 2.40% of the variance explained by a PRS; additively, the PRS and MRS accounted for 3.99% of variance explained in total. Although the PRS still outperforms the MRS on predictive ability, it is worth noting that PRS were trained on a sample of 807,579 individuals and the MRS on only 3047 individuals; although the proportion of variance explained by the MRS is currently small, the accuracy and clinical potential of MRS will likely increase as methylation data become more widely available. Therefore, MRS may yet provide clinically valuable information about the risk of future MDD. We found that MRS were associated with incident MDD, although they explained less of the variance in future MDD status (0.52%).

The MRS was associated with smoking status, pack years and alcohol consumption, suggesting that the MRS may reflect exposure to risky lifestyles known to be associated with MDD. After adjustment for lifestyle factors, the MRS association with MDD was substantially attenuated. These lifestyle factors have previously been associated with MDD [33–37] and are known to robustly associate with patterns of DNAm [12]. The attenuation of the association between MRS and MDD suggests that the DNAm-based predictor of MDD may be acting as a quantifiable archive of the longitudinal effects of these exposures, and other, lifestyle variables. Mediation analyses showed smoking significantly mediated the association between MDD and MRS, with 52.2% of the relationship being mediated by this lifestyle factor. This result is in line with other findings [8, 38], which indicate a strong influence of smoking on DNAm. In addition, the MRS was also significantly associated with self-reported antidepressant use, although this association does not account for the MRS-MDD associations reported. This finding suggests that MRS may also be sensitive to the effects of antidepressant use and that future studies should examine whether MRS trained on antidepressant use may be valuable as a measure of antidepressant absorption or pharmacological action.

Given the strong association between methylation status and smoking, we re-trained our MRS on a subset of cases and controls who had never smoked. The MRS-ns was still associated with MDD in the testing dataset but did explain less of the variance compared with the MRS (1.75% vs 0.4%). Surprisingly, the MRS-ns was still associated with smoking status and pack years but to a much lesser extent than the MRS. The MRS explained 3.2% of the variance in smoking status whereas the MRS-ns only explained 0.22% of the variance. Interestingly, the MRS-ns was now associated with BMI, a pattern not observed for the MRS. This suggests that when excluding the smoking signals from our dataset, the methylation differences between cases and controls were linked to BMI rather than smoking.

Correction for smoking status in case-control DNAm studies of other traits is an evolving area of methodological development. Correcting for current status alone tends to group together previous smokers and lifelong non-smokers, who may differ significantly in their smoking associated DNAm marks. Future studies may wish to correct for epigenetic smoking measures, as these are more stable and capture a larger proportion of smoking associated DNAm differences [39].

Although MRS were associated with exposure to environmental lifestyle factors, genetic effects are known to influence variation in CpG methylation status. Zeng et al. [5] recently found that SNP genetic effects explain, on average, 9.5% of the variance in methylation status at CpG sites across the genome and 7.2% of variance could be attributed to pedigree-associated effects. This was highly variable across the genome and only 24,101 CpG sites had statistically significant contributions from SNP genetic effects (*G*). The CpG sites comprising the MRS and the MRS-ns had higher contributions from SNP and pedigree-associated genetic effects than expected by chance. The proportion of variance explained by SNP effects for the CpG sites in the MRS was 15.1 and 16.5% for the MRS-ns. The additional pedigree-associated effects explained 9.7 and 14.5% of the variance in methylation for CpG sites making up the MRS and MRS-ns, respectively. Interestingly, 11 CpG sites in the MRS had mQTLs which were also strongly associated with MDD status in GWAS. Although the PRS derived from GWAS appear to be acting additively to the MRS risk for MDD, there are SNPs which associate with both CpG methylation and MDD risk. Future work should aim to determine whether these MDD-associated genetic variants influence risk for MDD via CpG methylation at these loci. There was little contribution from the shared family environment; however, when variance component analyses were applied to the total MRS, the shared couple environment significantly contributed to the variance explained. The couples in the GS:SFHS cohort are identified by shared probands and are likely to be co-habiting at the time of recruitment. The couple component therefore represents the recent shared environment and common exposure to lifestyle factors which influence DNAm. We recently showed there are strong couple environmental contributions to smoking and alcohol use which may explain why couples have similar MRS in the present sample [40].

Recent phenome-wide association studies have shown that MDD PRS are associated with a range of psychosocial and mental health phenotypes [16]. Using the same approach, we tested the association between 61 behavioural phenotypes and compared the pattern of association between MRS and PRS. MRS were significantly associated with cognition, lifestyle, physical and sociodemographic variables. In addition to these, PRS were associated with disease and mental health variables, such as MDD, number of depressive episodes and Mood Disorder Questionnaire score. The results indicate that PRS were associated with variables relating to MDD manifestation, as shown in previous studies [16]. Moreover, although both risk scores were associated with sociodemographic measures, such as years of education and deprivation ranks, MRS had a stronger association than PRS, indicating a stronger role played by the MRS in environmental factors [8–12].

Finally, pathway analysis conducted on the genes annotated to the two sets of CpG sites indicated that MRS annotated genes played a role in regulatory processes, while the MRS-ns annotated genes were enriched in neurodevelopmental processes within a large number of areas within the brain. These processes include axon guidance, neurogenesis and neuronal differentiation. This may show that excluding smoking signals from an MDD MRS may lead to uncovering locations along the genome which may play a role in neuronal processes.

The use of a single score instead of thousands of independent loci allows for a more comprehensive analysis investigating the additive effect of a large number of CpG sites and permits the use of smaller sample sizes. In the current study, we showed that an MRS could discriminate MDD cases and controls with an AUC of 0.58. Using machine learning methods and additional clinical variables, Clark et al. showed that this figure may be increased to 0.74 when investigating recurrent MDD cases only [13]. In this study, we were able to gain insight into a novel association between an MRS and depression, over and above genetic and environmental risk arising from lifestyle factors, as well as examine differences between MRS and PRS in various behavioural phenotypes. However, the MRS has less predictive ability for incident depression, indicating that the score performs better when assessing currently affected individuals. Moreover, although a proportion of the score seems to capture exposure to lifestyle factors, it is unclear what the remainder represents. As such, future studies would benefit from larger sample sizes and longitudinal samples to better understand the contribution of DNAm to MDD risk.

In addition, the diagnostic measures used in the current study differed at baseline (SCID) and follow-up (CIDI-SF). Previous evidence indicates that these two measures do not show perfect agreement [41]. Nevertheless, use of the CIDI-SF has been well-validated and has good diagnostic accuracy for MDD [42]. Future studies could usefully compare the DNAm profiles of MDD according to different diagnostic instruments.

In conclusion, our results show that an MRS is associated with current and future MDD status, enhancing prediction from PRS and environmental traits. Subsequent to further testing and validation in clinically-ascertained samples, these findings may have future clinical applications for MDD risk stratification and justify further efforts to collect DNAm in larger samples.

## Supplementary information

Supplementary materials

Supplementary file 1A

Supplementary file 1B

Supplementary file 2A

Supplementary file 2B
