# Supplementary material for: Epigenetic prediction of major depressive disorder
Source: Mol Psychiatry. 2020 Jun 10;26(9):5112–23. doi: 10.1038/s41380-020-0808-3 (PMC8589651; doi:10.1038/s41380-020-0808-3)
Supplement: Supplementary file 1 — Supplementary materials [file 41380_2020_808_MOESM1_ESM.docx]

**Supplementary materials**

**Baseline MDD in GS:SFHS**

The SCID was administered to participants who answered “yes” to either of the following screening questions: “Have you ever seen anybody for emotional or psychiatric problems?” and “Was there ever a time when you, or someone else, thought you should see someone because of the way you were feeling or acting?”. Answers from the SCID were used to ascertain MDD case status.

**Antidepressant use measurement in GS:SFHS**

A self-report measure of antidepressant use was recorded by participants in two different ways: within the first phase of the study, a text-based questionnaire was used to record type of antidepressant taken; participants recruited between June 2009 and March 2011 completed a questionnaire recording medication use through a “yes/no” checkbox, with an accompanying question: “Are you regularly taking any of the following medications?”, of which one of the answers was “Antidepressants”. The total number of individuals reporting antidepressant use in both the training and testing datasets is reported in Supplementary Tables 1 and 2.

**R code (created using R Markdown)**

DNAm analysis

Miruna Barbu

## Association of MRS and MRS-ns with depression

# Read in datasets
#### PREVALENCE ####
MRS_data_prevalence = readRDS("*/path-to-file/file.rds* ")

# Data analysis
glm_prev = glm(dep_both ~ age+sex+C1+C2+C3+C4+C5+C6+C7+C8+C9+C10+ DNAm_predictor_prevalence,family=binomial,data = MRS_data_prevalence)

prevalence = print(summary(glm_prev))

##
## Call:
## glm(formula = dep_both ~ age + sex + C1 + C2 + C3 + C4 + C5 +
## C6 + C7 + C8 + C9 + C10 + DNAm_predictor_prevalence, family = binomial,
## data = MRS_data_prevalence)
##
## Deviance Residuals:
## Min 1Q Median 3Q Max
## -1.7388 -0.6815 -0.5071 -0.3217 2.3697
##
## Coefficients:
## Estimate Std. Error z value Pr(>|z|)
## (Intercept) 1.638231 0.346994 4.721 2.34e-06 ***
## age -0.049321 0.005275 -9.350 < 2e-16 ***
## sexM -0.793273 0.132902 -5.969 2.39e-09 ***
## C1 -43.574203 45.401646 -0.960 0.33718
## C2 22.607276 47.261872 0.478 0.63241
## C3 -1.816845 7.713746 -0.236 0.81379
## C4 32.702042 10.891643 3.002 0.00268 **
## C5 -30.253992 13.959165 -2.167 0.03021 *
## C6 -9.907077 11.747573 -0.843 0.39904
## C7 -32.461335 22.550957 -1.439 0.15002
## C8 73.351816 27.879508 2.631 0.00851 **
## C9 36.986127 15.680258 2.359 0.01834 *
## C10 -1.088721 15.890232 -0.069 0.94538
## DNAm_predictor_prevalence 0.337608 0.063726 5.298 1.17e-07 ***
## ---
## Signif. codes: 0 '***' 0.001 '**' 0.01 '*' 0.05 '.' 0.1 ' ' 1
##
## (Dispersion parameter for binomial family taken to be 1)
##
## Null deviance: 1800.7 on 1779 degrees of freedom
## Residual deviance: 1613.9 on 1766 degrees of freedom
## AIC: 1641.9
##
## Number of Fisher Scoring iterations: 4

# Calculate R2 for logistic regression
nullmod <- glm(dep_both ~ age+sex+C1+C2+C3+C4+C5+C6+C7+C8+C9+C10,data = MRS_data_prevalence, family="binomial")
1-logLik(glm_prev)/logLik(nullmod)

## 'log Lik.' 0.01749893 (df=14)

## INCIDENCE ####
MRS_data_incidence = readRDS("*/path-to-file/file.rds* ")

# Data analysis
glm_inc = glm(dep_develop ~ age+sex+C1+C2+C3+C4+C5+C6+C7+C8+C9+C10+ DNAm_predictor_incidence,family=binomial,data = MRS_data_incidence)

incidence=print(summary(glm_inc))

##
## Call:
## glm(formula = dep_develop ~ age + sex + C1 + C2 + C3 + C4 + C5 +
## C6 + C7 + C8 + C9 + C10 + DNAm_predictor_incidence, family = binomial,
## data = MRS_data_incidence)
##
## Deviance Residuals:
## Min 1Q Median 3Q Max
## -0.9756 -0.5428 -0.4495 -0.3542 2.5964
##
## Coefficients:
## Estimate Std. Error z value Pr(>|z|)
## (Intercept) -0.097095 0.445452 -0.218 0.8275
## age -0.026874 0.006792 -3.957 7.59e-05 ***
## sexM -0.681103 0.166461 -4.092 4.28e-05 ***
## C1 -60.778710 54.967716 -1.106 0.2688
## C2 -40.261187 60.026028 -0.671 0.5024
## C3 -6.628326 9.389932 -0.706 0.4803
## C4 23.082692 13.638374 1.692 0.0906 .
## C5 -9.431667 17.320876 -0.545 0.5861
## C6 -14.785982 14.859521 -0.995 0.3197
## C7 6.905293 27.699192 0.249 0.8031
## C8 -15.772336 34.361569 -0.459 0.6462
## C9 28.373211 20.108518 1.411 0.1582
## C10 9.835888 20.354263 0.483 0.6289
## DNAm_predictor_incidence 0.193437 0.080121 2.414 0.0158 *
## ---
## Signif. codes: 0 '***' 0.001 '**' 0.01 '*' 0.05 '.' 0.1 ' ' 1
##
## (Dispersion parameter for binomial family taken to be 1)
##
## Null deviance: 1167.9 on 1606 degrees of freedom
## Residual deviance: 1120.2 on 1593 degrees of freedom
## AIC: 1148.2
##
## Number of Fisher Scoring iterations: 5

# Calculate R2 for logistic regression
nullmod <- glm(dep_develop ~ age+sex+C1+C2+C3+C4+C5+C6+C7+C8+C9+C10,data = MRS_data_incidence, family="binomial")
1-logLik(glm_inc)/logLik(nullmod)

## 'log Lik.' 0.005193741 (df=14)

# For both incident and prevalent MDD, 4 lifestyle factors (BMI, alcohol consumption, smoking status, pack years) and self-reported antidepressant use were added into statistical models, with MRS included to compare the additional variance explained by the risk score

# All above analyses were carried out with the methylation risk score trained on non-smokers only (MRS-ns)

knitr::opts_chunk$set(echo = TRUE)

## Association of MRS and MRS-ns with lifestyle factors

##### PREVALENCE ####
# BMI

lm_prev_bmi = lm(bmi ~ DNAm_predictor_prevalence,data = MRS_data_prevalence)
bmi = print(summary(lm_prev_bmi))

##
## Call:
## lm(formula = bmi ~ DNAm_predictor_prevalence, data = MRS_data_prevalence)
##
## Residuals:
## Min 1Q Median 3Q Max
## -2.1502 -0.6585 -0.1591 0.4919 4.8625
##
## Coefficients:
## Estimate Std. Error t value Pr(>|t|)
## (Intercept) -0.01448 0.02321 -0.624 0.5327
## DNAm_predictor_prevalence 0.03855 0.02333 1.653 0.0985 .
## ---
## Signif. codes: 0 '***' 0.001 '**' 0.01 '*' 0.05 '.' 0.1 ' ' 1
##
## Residual standard error: 0.979 on 1778 degrees of freedom
## Multiple R-squared: 0.001534, Adjusted R-squared: 0.0009725
## F-statistic: 2.732 on 1 and 1778 DF, p-value: 0.09855

# Alcohol consumption

lm_prev_alc = lm(units ~ DNAm_predictor_prevalence,data = MRS_data_prevalence)
alc = print(summary(lm_prev_alc))

##
## Call:
## lm(formula = units ~ DNAm_predictor_prevalence, data = MRS_data_prevalence)
##
## Residuals:
## Min 1Q Median 3Q Max
## -1.2075 -0.6597 -0.2400 0.3955 11.7079
##
## Coefficients:
## Estimate Std. Error t value Pr(>|t|)
## (Intercept) -0.001601 0.023558 -0.068 0.946
## DNAm_predictor_prevalence 0.092427 0.023681 3.903 9.85e-05 ***
## ---
## Signif. codes: 0 '***' 0.001 '**' 0.01 '*' 0.05 '.' 0.1 ' ' 1
##
## Residual standard error: 0.9938 on 1778 degrees of freedom
## Multiple R-squared: 0.008495, Adjusted R-squared: 0.007937
## F-statistic: 15.23 on 1 and 1778 DF, p-value: 9.853e-05

# Smoking status

glm_prev_smoke = glm(ever_smoke ~ DNAm_predictor_prevalence,family=binomial,data = MRS_data_prevalence)
smoke = print(summary(glm_prev_smoke))

##
## Call:
## glm(formula = ever_smoke ~ DNAm_predictor_prevalence, family = binomial,
## data = MRS_data_prevalence)
##
## Deviance Residuals:
## Min 1Q Median 3Q Max
## -1.818 -1.072 -0.856 1.185 1.971
##
## Coefficients:
## Estimate Std. Error z value Pr(>|z|)
## (Intercept) -0.18449 0.04868 -3.790 0.000151 ***
## DNAm_predictor_prevalence 0.43982 0.05115 8.598 < 2e-16 ***
## ---
## Signif. codes: 0 '***' 0.001 '**' 0.01 '*' 0.05 '.' 0.1 ' ' 1
##
## (Dispersion parameter for binomial family taken to be 1)
##
## Null deviance: 2452.8 on 1779 degrees of freedom
## Residual deviance: 2373.9 on 1778 degrees of freedom
## AIC: 2377.9
##
## Number of Fisher Scoring iterations: 4

# Calculate R2 for logistic regression
nullmod <- glm(ever_smoke ~ 1,data = MRS_data_prevalence, family="binomial")
1-logLik(glm_prev_smoke)/logLik(nullmod)

## 'log Lik.' 0.03219957 (df=2)

# Pack years

lm_prev_pack_yrs = lm(pack_years ~ DNAm_predictor_prevalence,data = MRS_data_prevalence)
pack_yrs = print(summary(lm_prev_pack_yrs))

##
## Call:
## lm(formula = pack_years ~ DNAm_predictor_prevalence, data = MRS_data_prevalence)
##
## Residuals:
## Min 1Q Median 3Q Max
## -1.4038 -0.5108 -0.3140 0.0948 6.8330
##
## Coefficients:
## Estimate Std. Error t value Pr(>|t|)
## (Intercept) -0.01990 0.02184 -0.911 0.362
## DNAm_predictor_prevalence 0.24581 0.02195 11.197 <2e-16 ***
## ---
## Signif. codes: 0 '***' 0.001 '**' 0.01 '*' 0.05 '.' 0.1 ' ' 1
##
## Residual standard error: 0.9213 on 1778 degrees of freedom
## Multiple R-squared: 0.06587, Adjusted R-squared: 0.06535
## F-statistic: 125.4 on 1 and 1778 DF, p-value: < 2.2e-16

# All above analyses were carried out with the methylation risk score calculated for incident depression

# All above analyses were carried out with the methylation risk score trained on non-smokers only (MRS-ns)

|  | **MDD diagnosis** | **No MDD diagnosis** |
| --- | --- | --- |
| **MRS (smokers & non-smokers) N_total_ = 3,047; N_cases_ = 1,223** |  |  |
| Age (Mean +/- SD, range) | 48.44 +/- 12.07, 18 - 82 | 50.36 +/- 13.40, 18 - 95 |
| Sex |  |  |
| Female | 881 | 1059 |
| Male | 342 | 765 |
| BMI (Mean +/- SD, range) | 27.51 +/- 5.95, 14.58 – 56.60 | 26.83 +/- 5.02, 16.14 – 54.64 |
| Alcohol units (Mean +/- SD, range) | 10.25 +/- 11.88, 0 - 105 | 10.4 +/- 12, 0 - 128 |
| Smoking status |  |  |
| Yes | 659 | 779 |
| No | 535 | 1,007 |
| Pack years (Mean +/- SD, range) | 8.57 +/- 13.93, 0 – 120 | 7.38 +/- 14.56, 0 – 107.6 |
| Current self-reported antidepressant use |  |  |
| Yes | 390 | 61 |
| No | 618 | 1,302 |
| **MRS-ns (non-smokers) N_total_ = 1,551; N_cases_ = 534** |  |  |
| Age (Mean +/- SD, range) | 47.8 +/- 12.71, 18 - 82 | 49.08 +/- 13.90, 18 - 95 |
| Sex |  |  |
| Female | 403 | 629 |
| Male | 131 | 388 |
| BMI (Mean +/- SD, range) | 27.51 +/- 5.98, 15.25 – 56.60 | 26.47 +/- 5.04, 16.14 – 54.64 |
| Alcohol units (Mean +/- SD, range) | 9.04 +/- 10.55, 0 - 84 | 8.75 +/- 10.40, 0 - 108 |
| Current self-reported antidepressant use |  |  |
| Yes | 156 | 25 |
| No | 290 | 746 |

**Supplementary Table 1.** Demographic characteristics for the individuals in the training samples for MRS and MRS-ns. Smoking information is not available for the MRS-ns, as only non-smokers were selected.

|  | **MDD diagnosis** | **No MDD diagnosis** |
| --- | --- | --- |
| **Prevalence dataset (N_total_ = 1,780, N_cases_ = 363)** |  |  |
| Age (Mean +/- SD, range) | 47.46 +/- 11.99, 18 – 74 | 54.35 +/- 11.05, 18 – 83 |
| Sex |  |  |
| Female | 258 | 730 |
| Male | 105 | 687 |
| BMI (Mean +/- SD, range) | 27.08 +/- 5.42, 16.46 – 47.30 | 26.24 +/- 4.40, 16.72 – 49.58 |
| Alcohol units (Mean +/- SD, range) | 10.93 +/- 13.99, 0 – 146 | 10.74 +/- 10.71, 0 – 100 |
| Smoking status |  |  |
| Yes | 208 | 589 |
| No | 151 | 820 |
| Pack years (Mean +/- SD, range) | 10.02 +/- 16.12, 0 - 84 | 6.09 +/- 12.38, 0 - 102 |
| Current self-reported antidepressant use |  |  |
| Yes | 108 | 27 |
| No | 198 | 1,052 |
| **Incidence dataset (N_total_ = 1,607; N_cases_ = 190)** |  |  |
| Age (Mean +/- SD, range) | 50.85 +/- 10.95, 20 – 81 | 54.35 +/- 11.05, 18 – 83 |
| Sex |  |  |
| Female | 129 | 730 |
| Male | 61 | 687 |
| BMI (Mean +/- SD, range) | 26.73 +/- 4.85, 17.67 – 45.02 | 26.24 +/- 4.40, 16.72 – 49.58 |
| Alcohol units (Mean +/- SD, range) | 9.21 +/- 10.18, 0 – 72 | 10.74 +/- 10.71, 0 – 100 |
| Smoking status |  |  |
| Yes | 107 | 589 |
| No | 83 | 820 |
| Pack years (Mean +/- SD, range) | 7.6 +/- 12.19, 0 – 68.45 | 6.09 +/- 12.38, 0 – 102 |
| Current self-reported antidepressant use |  |  |
| Yes | 20 | 27 |
| No | 143 | 1,052 |

**Supplementary Table 2.** Demographic characteristics for individuals in prevalence and incidence testing samples.

**
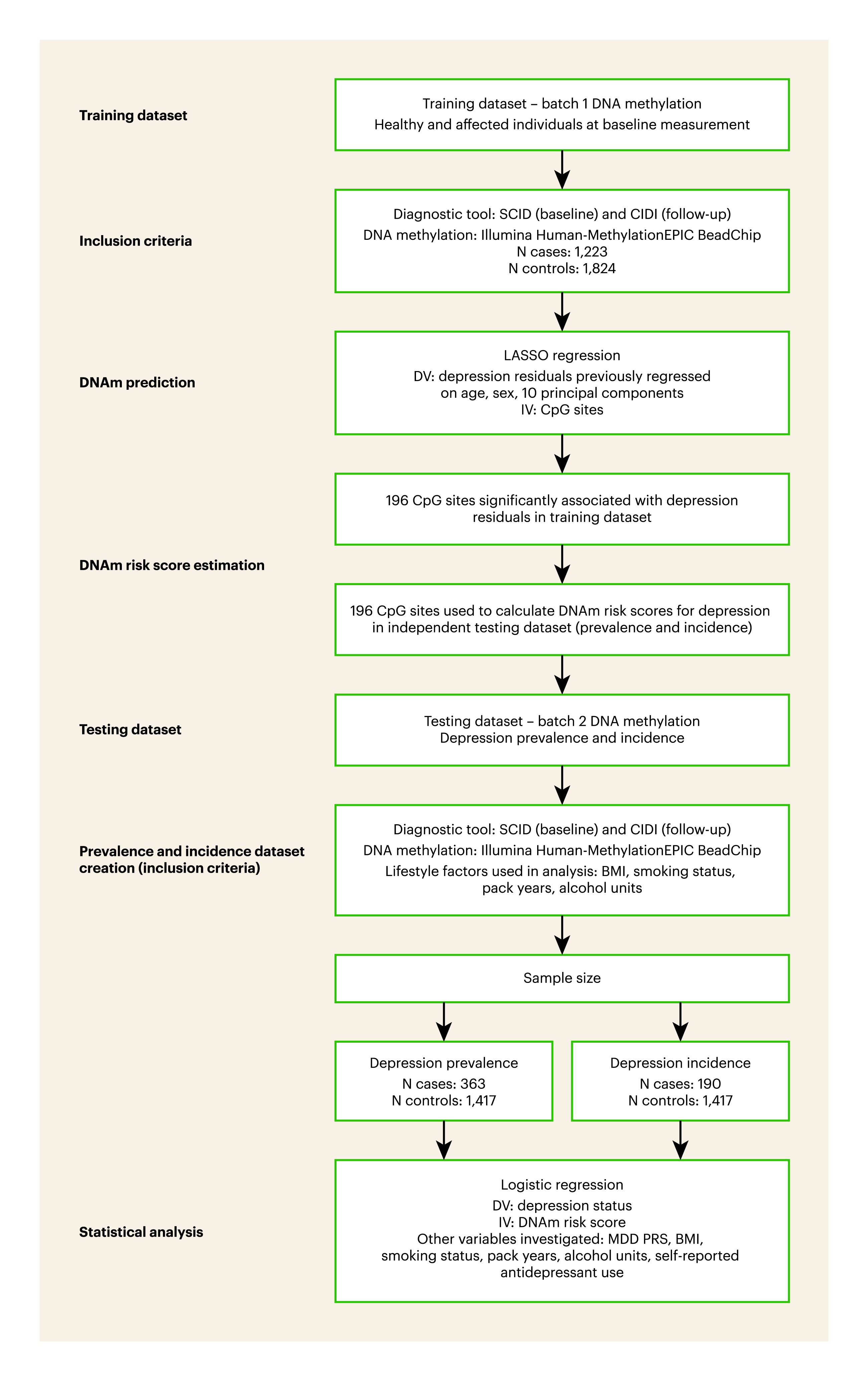
Supplementary Figure 1.** Flowchart indicating the analysis process in training and testing datasets.

| **Category** | **Description** | **MRS N** | **PRS N** |
| --- | --- | --- | --- |
| Cognition | Digit-Symbol Coding Total Correct | 1762 | 1717 |
| Cognition | Logical Memory Total | 1780 | 1735 |
| Cognition | Verbal Fluency Total | 1768 | 1723 |
| Cognition | Mill Hill Vocabulary Total Correct | 1759 | 1714 |
| Disease | Asthma | 1102 | 1071 |
| Disease | Bowel Cancer | 1102 | 1071 |
| Disease | Breast Cancer | 1102 | 1071 |
| Disease | COPD | 1102 | 1071 |
| Disease | Depression | 1102 | 1071 |
| Disease | Diabetes | 1102 | 1071 |
| Disease | Heart Disease | 1102 | 1071 |
| Disease | High Blood Pressure | 1102 | 1071 |
| Disease | Hip Fracture | 1102 | 1071 |
| Disease | Lung Cancer | 1102 | 1071 |
| Disease | Osteoarthritis | 1102 | 1071 |
| Disease | Other serious illness | 1102 | 1071 |
| Disease | Parkinson's Disease | 1102 | 1071 |
| Disease | Prostate Cancer | 1102 | 1071 |
| Disease | Rheumatoid Arthritis | 1102 | 1071 |
| Disease | Stroke | 1102 | 1071 |
| Lifestyle measure | Age started smoking | 791 | 777 |
| Lifestyle measure | Daily cigarette smoking | 732 | 718 |
| Lifestyle measure | Ever smoked tobacco | 1768 | 1723 |
| Lifestyle measure | Hours per week exposed to tobacco smoke | 1648 | 1607 |
| Lifestyle measure | Years stopped smoking | 573 | 561 |
| Lifestyle measure | Alcohol consumption (units/last week) | 1780 | 1735 |
| Mental health/Psychology | SCID-depressive status | 1780 | 1735 |
| Mental health/Psychology | Number of depressive episodes | 1780 | 1735 |
| Mental health/Psychology | Extraversion | 1653 | 1613 |
| Mental health/Psychology | GHQ-Total (likert) | 1764 | 1719 |
| Mental health/Psychology | Depression (single vs recurrent) | 1780 | 1735 |
| Mental health/Psychology | MDQ (Mood Disorder Ques) | 1066 | 1037 |
| Mental health/Psychology | Neuroticism | 1653 | 1613 |
| Mental health/Psychology | Schizophrenic symptoms (SPQ) | 1107 | 1076 |
| Physical measure | Mean diastolic blood pressure | 1778 | 1733 |
| Physical measure | Mean heart rate | 1777 | 1732 |
| Physical measure | Mean systolic blood pressure | 1778 | 1733 |
| Physical measure | Body Mass Index | 1780 | 1735 |
| Physical measure | Body Fat Composition (bio-impedance) | 1758 | 1715 |
| Physical measure | Creatinine mg/dL | 1775 | 1730 |
| Physical measure | Glucose | 1748 | 1705 |
| Physical measure | HDL cholesterol | 1771 | 1727 |
| Physical measure | Hypertension (from blood pressure) | 1778 | 1733 |
| Physical measure | Maximum systolic blood pressure | 1778 | 1733 |
| Physical measure | Potassium | 1744 | 1702 |
| Physical measure | Sodium | 1775 | 1730 |
| Physical measure | Total cholesterol | 1772 | 1728 |
| Physical measure | Urea | 1775 | 1730 |
| Physical measure | Waist / Hip Ratio | 1764 | 1720 |
| Sociodemographic | Living with a partner | 1755 | 1712 |
| Sociodemographic | Number of people in the household | 1767 | 1724 |
| Sociodemographic | Accommodation status (own<rent) | 1764 | 1720 |
| Sociodemographic | Accommodation type (house < homeless) | 1774 | 1730 |
| Sociodemographic | Number of vehicles available (household) | 1764 | 1721 |
| Sociodemographic | Average household income | 1643 | 1603 |
| Sociodemographic | Years of joblessness | 990 | 966 |
| Sociodemographic | Hours working in the evening/week | 1166 | 1136 |
| Sociodemographic | Deprivation ranks (SIMD) | 1712 | 1670 |
| Sociodemographic | Job status of spouse | 1523 | 1486 |
| Sociodemographic | Job status of oneself | 1626 | 1586 |
| Sociodemographic | Years of education | 1729 | 1684 |

**Supplementary Table 3**. Phenotypes included as outcome variables in analyses including MRS and PRS as predictors.

| **Statistic** | **IV: DNAm** | **IV: PRS** | **IV: PRS + DNAm** |
| --- | --- | --- | --- |
| **Effect size, β** |  |  |  |
| DNAm | 0.338 | - | 0.327 |
| PRS | - | 0.397 | 0.384 |
| **SD** |  |  |  |
| DNAm | 0.064 | - | 0.065 |
| PRS | - | 0.065 | 0.066 |
| **t value** |  |  |  |
| DNAm | 5.289 | - | 5.002 |
| PRS | - | 6.107 | 5.858 |
| **Nominal p value** |  |  |  |
| DNAm | 1.17x10^-7^ | - | 5.66x10^-7^ |
| PRS | - | 1.02x10^-9^ | 4.69x10^-9^ |
| **R^2^** | 1.75% | 2.40% | 3.99% |

**Supplementary Table 4**. Associations between MRS, PRS, and PRS + MRS (included as predictor variables) and MDD (included as the outcome variable). Results in the table include standardised effect size, standard error (SD), t value, nominal p-value, and R^2^ (%) comparing the three models (MRS, PRS, and PRS + MRS); IV = independent variable included in the model.

| **Lifestyle factor** | **Effect size** | **SD** | **t value** | **p value** | **R^2^** |
| --- | --- | --- | --- | --- | --- |
| **MRS** |  |  |  |  |  |
| BMI | 0.039 | 0.023 | 1.653 | 0.099 | 0.097% |
| Smoking status | 0.440 | 0.051 | 8.598 | < 2x10^-16^ | 3.2% |
| Pack years | 0.246 | 0.022 | 11.197 | < 2x10^-16^ | 6.5% |
| Alcohol units | 0.092 | 0.024 | 3.903 | 9.85x10^-5^ | 0.7% |
| Self-report antidepressant use  (N = 1385) | 0.289 | 0.091 | 3.165 | 0.002 | 1.1% |
| **MRS-ns** |  |  |  |  |  |
| BMI | 0.053 | 0.023 | 2.318 | 0.021 | 0.246% |
| Smoking status | 0.102 | 0.048 | 2.107 | 0.035 | 0.224% |
| Pack years | 0.055 | 0.022 | 2.462 | 0.014 | 0.272% |
| Alcohol units | 0.024 | 0.022 | 1.062 | 0.289 | 0.01% |
| Self-report antidepressant use  (N = 1385) | 0.084 | 0.093 | 0.906 | 0.365 | 0.096% |

**Supplementary Table 5**. Associations between lifestyle factors (BMI, smoking status, pack years, and alcohol units), self-reported antidepressant use, and MRS and MRS-ns. Results include standardised effect size, standard error (SD), t value, nominal p-value and variance explained (R^2^) in the four lifestyle factors (N = 1,780) and self-report antidepressant use (N = 1,385) by the two risk scores.


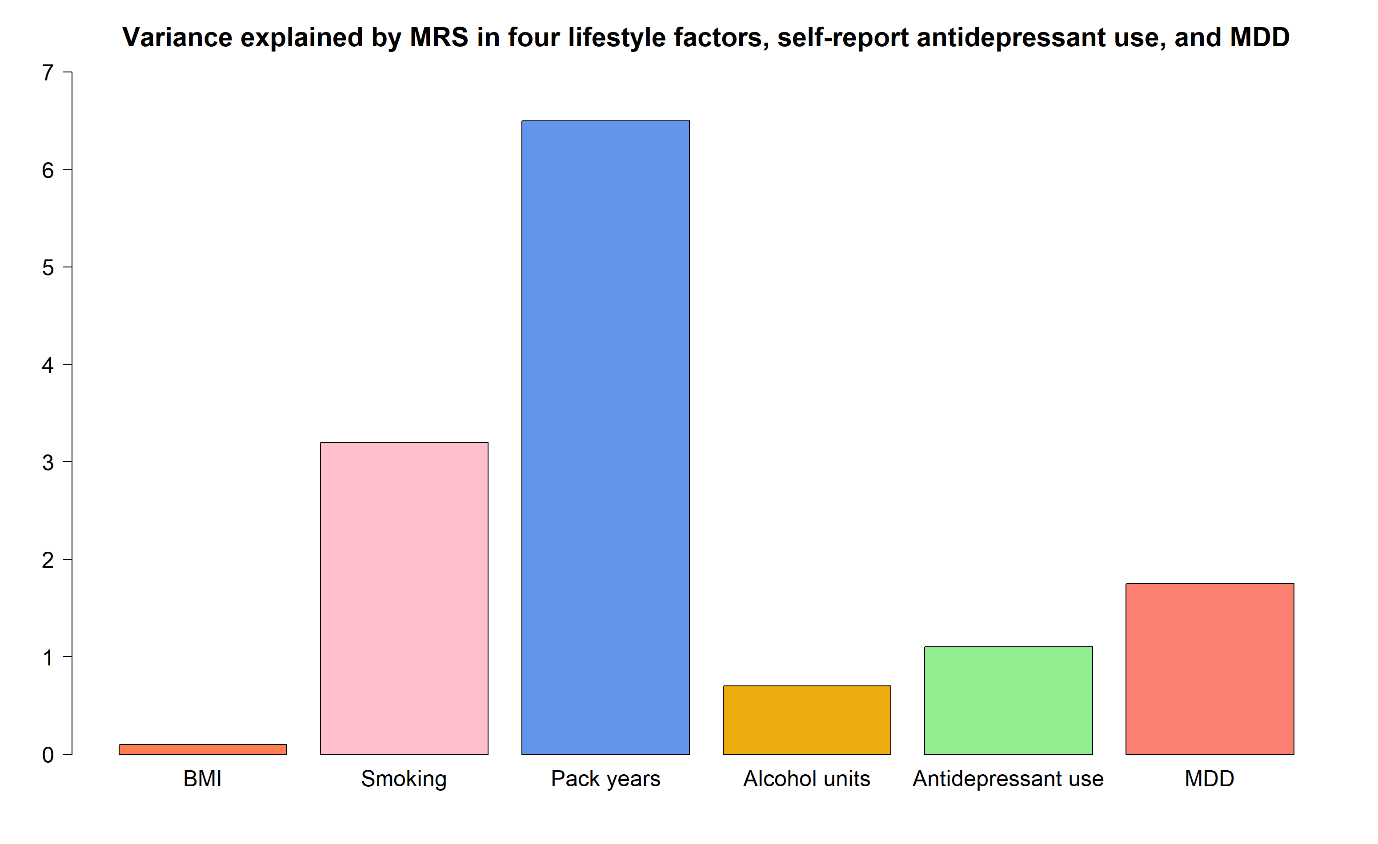


**Supplementary Figure 2.** Variance in BMI (coral), smoking status (pink), pack years (blue), alcohol units (yellow), self-reported antidepressant use (green) and prevalent MDD (salmon) (indicated by R^2^ (%) on the y-axis) explained by MRS; N = 1,780 for all x-axis variables except self-reported antidepressant use (N = 1,385).


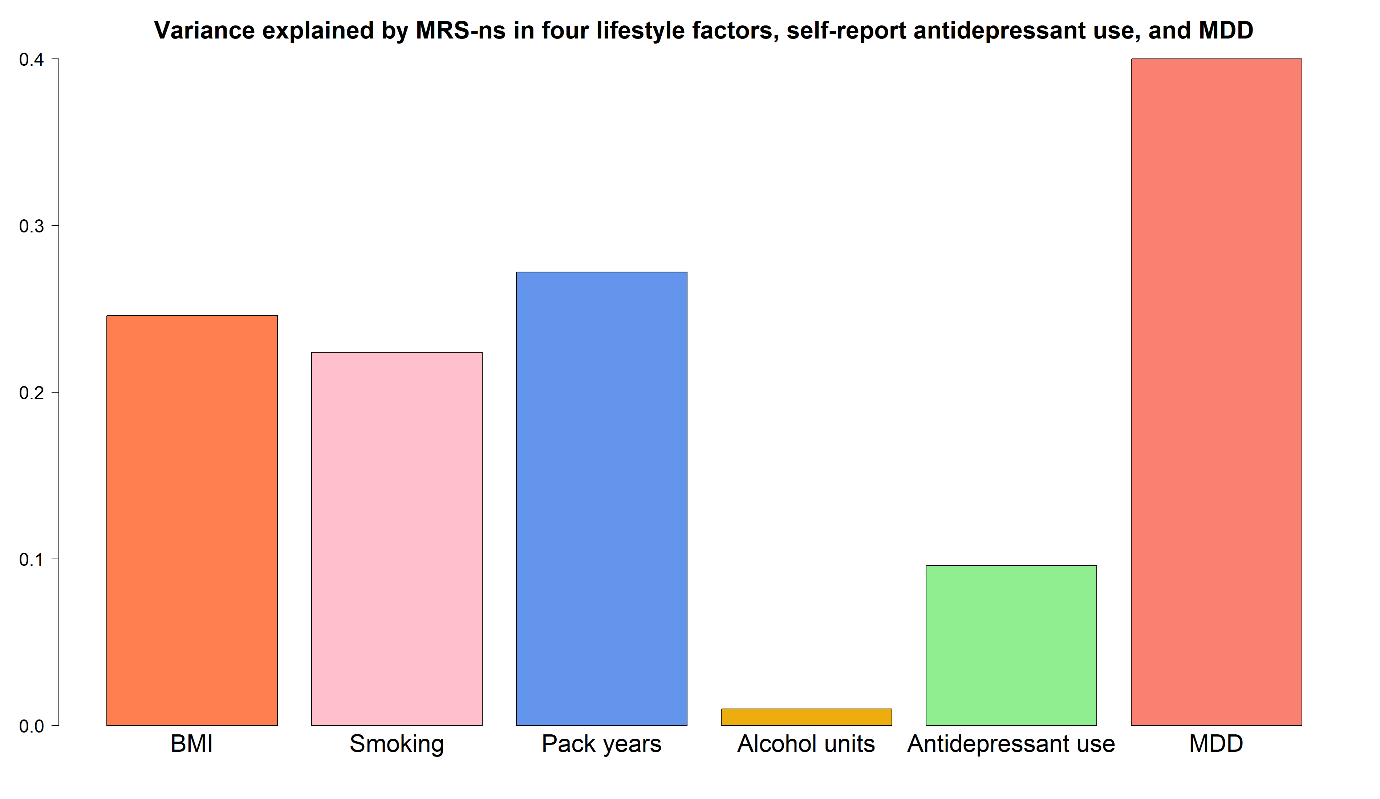


**Supplementary Figure 3.** Variance in BMI (coral), smoking status (pink), pack years (blue), alcohol units (yellow), self-reported antidepressant use (green) and prevalent MDD (salmon) (indicated by R^2^ (%) on the y-axis) explained by MRS-ns; N = 1,780 for all x-axis variables except self-reported antidepressant use (N = 1,385).


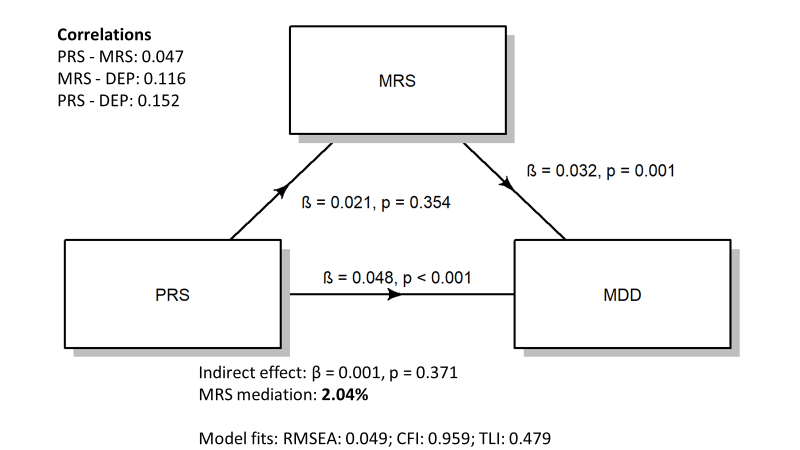

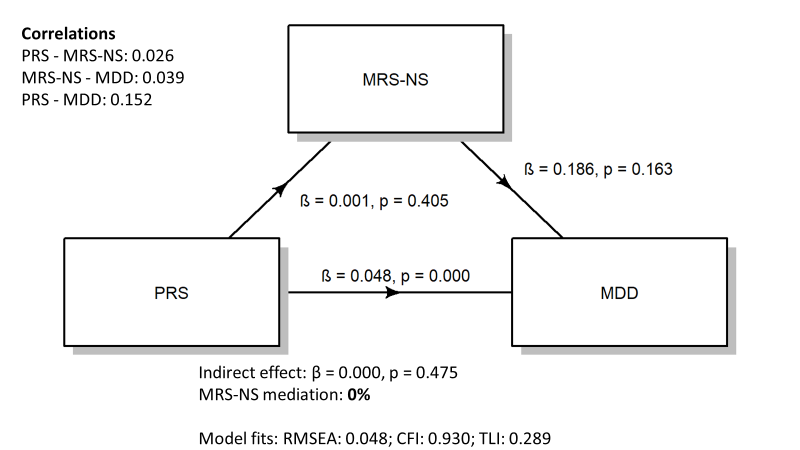


**Supplementary Figure 4**. Mediation analysis including MRS as a mediator between PRS and MDD.


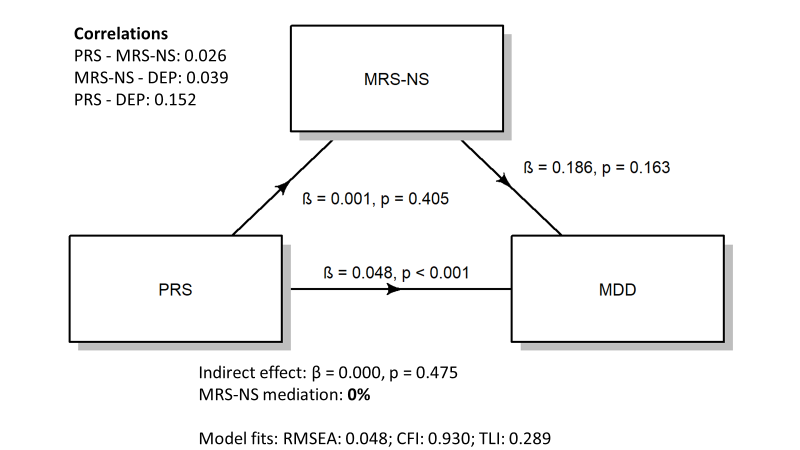


**Supplementary Figure 5**. Mediation analysis including MRS-ns as a mediator between PRS and MDD.

| **CpG** | **Chr** | **Locus** | **Top mQTL** | **Additional**  **mQTL** | **Meth effect** | **Meth pvalue** | **Dep effect** | **Dep pvalue** | **Dep FDR** |
| --- | --- | --- | --- | --- | --- | --- | --- | --- | --- |
| cg00318111 | 7 | *RSPH10B* | rs10272684 | NA | 0.004 | 5.3E-08 | -0.014 | 1.6E-03 | 2.8E-02 |
| cg08754268 | 2 | *RAPGEF4* | rs12986495 | 66 | 0.063 | 7.3E-80 | 0.014 | 1.4E-04 | 3.0E-03 |
| cg09333631 | 3 | *KIAA1143* | rs9826486 | 388 | 0.022 | 8.4E-21 | 0.017 | 2.5E-06 | 9.1E-05 |
| cg15611176 | 12 | *PLA2G1B* | rs1179450 | 7 | 0.001 | 8.5E-13 | 0.013 | 3.9E-04 | 7.9E-03 |
| cg17916960 | 15 | *ANKRD34C-AS1* | rs4778886 | NA | 0.103 | 4.3E-45 | 0.012 | 2.2E-03 | 3.8E-02 |
| cg20475550 | 10 | *PPP1R3C* | rs1891275 | 19 | 0.009 | 2.2E-12 | 0.016 | 7.4E-06 | 2.4E-04 |
| cg21033440 | 11 | *SIPA1* | rs4930319 | NA | 0.027 | 5.5E-10 | 0.011 | 3.2E-03 | 4.9E-02 |
| cg24147428 | 11 | *SIPA1* | rs4930319 | NA | 0.024 | 1.7E-10 | 0.011 | 3.2E-03 | 4.9E-02 |
| cg26090062 | 6 | *PRSS16* | rs9468014 | 19 | 0.099 | 1.6E-08 | -0.015 | 1.8E-03 | 3.1E-02 |
| cg26872792 | 12 | *PLA2G1B* | rs1179434 | 7 | 0.000 | 8.4E-10 | -0.012 | 5.4E-04 | 1.0E-02 |
| cg27332938 | 10 | *ZNF511/TUBGCP2* | rs2995334 | 20 | 0.016 | 3.3E-68 | 0.019 | 2.1E-04 | 4.4E-03 |

**Supplementary Table 6. mQTLs for CpGs comprising the MRS and their association with MDD.** Five hundred and thirty-six mQTLs displayed significant association with MDD after FDR correction and these were associated with 11 CpGs displayed here. Top mQTL shows the SNP with the strongest association with CpG methylation in the ARIES database. Meth effect and pvalue pertain to the association between mQTL and CpG methylation, Dep effect, pvalue and FDR show the association of the mQTL with MDD and the pvalue after correction for multiple testing and were obtained from GWAS summary statistics (4).

| **Outcome variable** | **Category** | **Predictor variable** | **Effect Size, β** | **SD** | **t value** | **p value** | **p_corrected_** |
| --- | --- | --- | --- | --- | --- | --- | --- |
| Digit-Symbol Coding Total Correct | Cognition | MRS | -0.068 | 0.022 | -3.133 | 0.0018 | 0.011 |
| Mill Hill Vocabulary Total Correct | Cognition | MRS | -0.078 | 0.023 | -3.437 | 0.0006 | 0.005 |
| Years stopped smoking | Lifestyle measure | MRS | -0.122 | 0.037 | -3.326 | 0.0009 | 0.006 |
| Potassium | Physical measure | MRS | 0.073 | 0.024 | 3.037 | 0.002 | 0.013 |
| Waist / Hip Ratio | Physical measure | MRS | 0.05 | 0.019 | 2.636 | 0.008 | 0.037 |
| Accommodation status (own<rent) | Sociodemographic | MRS | 0.060 | 0.022 | 2.811 | 0.004 | 0.025 |
| Deprivation ranks (SIMD) | Sociodemographic | MRS | -0.081 | 0.024 | -3.418 | 0.0006 | 0.005 |
| Years of education | Sociodemographic | MRS | -0.103 | 0.024 | -4.338 | 0.000015 | 0.0002 |
| Digit-Symbol Coding Total Correct | Cognition | PRS 0.05 | -0.085 | 0.022 | -3.896 | 0.0001 | 0.0009 |
| Depression | Disease | PRS 0.05 | 0.097 | 0.029 | 3.314 | 0.0009 | 0.006 |
| Daily cigarette smoking | Lifestyle measure | PRS 0.05 | 0.097 | 0.038 | 2.569 | 0.0104 | 0.044 |
| Ever smoked tobacco | Lifestyle measure | PRS 0.05 | -0.065 | 0.024 | -2.709 | 0.007 | 0.031 |
| Number of depressive episodes | Mental health/Psychology | PRS 0.05 | 0.082 | 0.024 | 3.431 | 0.00062 | 0.005 |
| MDQ (Mood Disorder Ques) | Mental health/Psychology | PRS 0.05 | 0.088 | 0.030 | 2.936 | 0.0034 | 0.018 |
| Body Mass Index | Physical measure | PRS 0.05 | 0.073 | 0.024 | 3.076 | 0.0021 | 0.012 |
| Body Fat Composition (bio-impedance) | Physical measure | PRS 0.05 | 0.052 | 0.019 | 2.749 | 0.006 | 0.029 |
| Deprivation ranks (SIMD) | Sociodemographic | PRS 0.05 | -0.085 | 0.024 | -3.564 | 0.0004 | 0.003 |
| Years of education | Sociodemographic | PRS 0.05 | -0.066 | 0.024 | -2.733 | 0.0063 | 0.029 |

**Supplementary Table 7**. MRS and PRS were included as predictors for the outcome variables specified in the table in regression analyses; results include standardised effect size, standard error (SD), t-value, p-value, and p_corrected_.


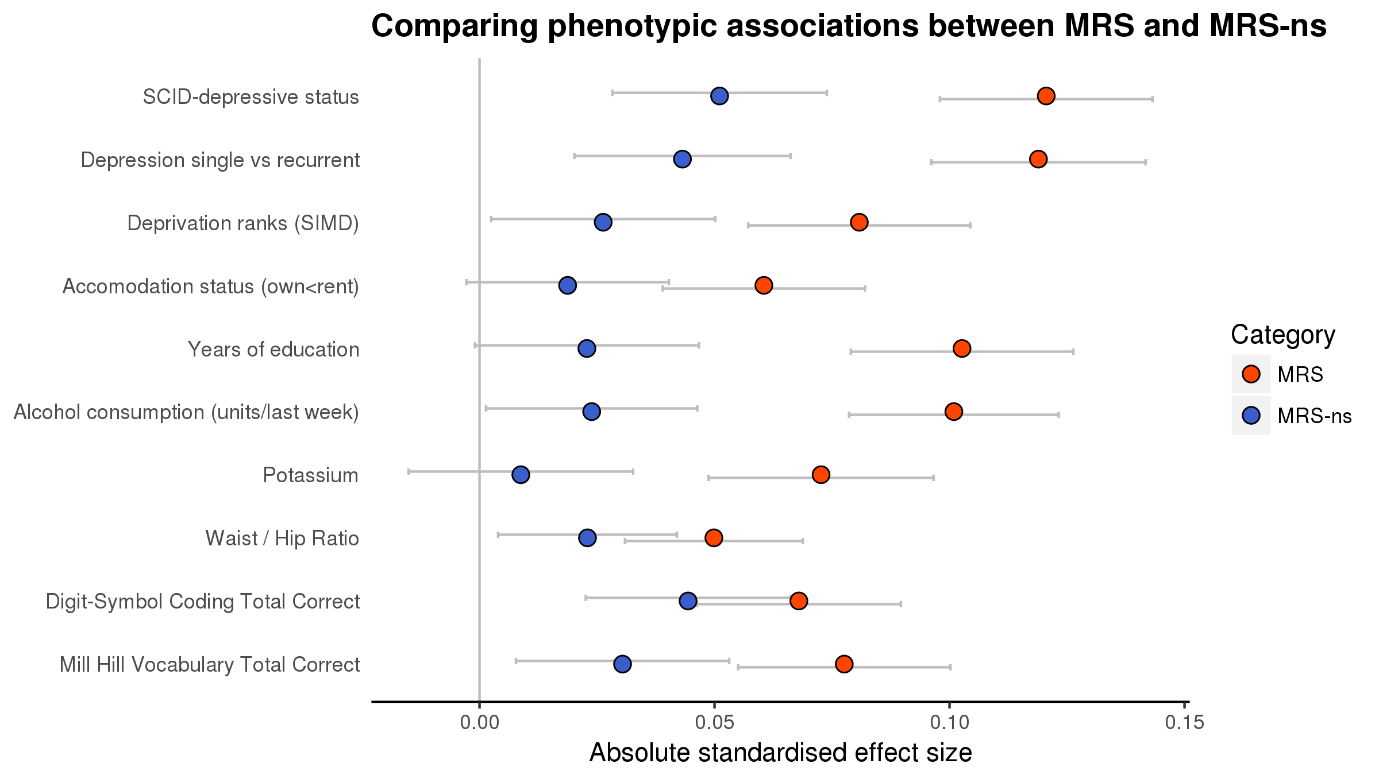


**Supplementary Figure 6.** Comparison of MRS and MRS-ns (trained on non-smokers) for outcome variables outlined on the y-axis; x-axis contains absolute standardised effect sizes for each outcome variable. Error bars represent standard errors of the effect size.

**Pathway analysis – Gene Ontology gene sets with enrichment p-values for each gene**

**MRS**

**
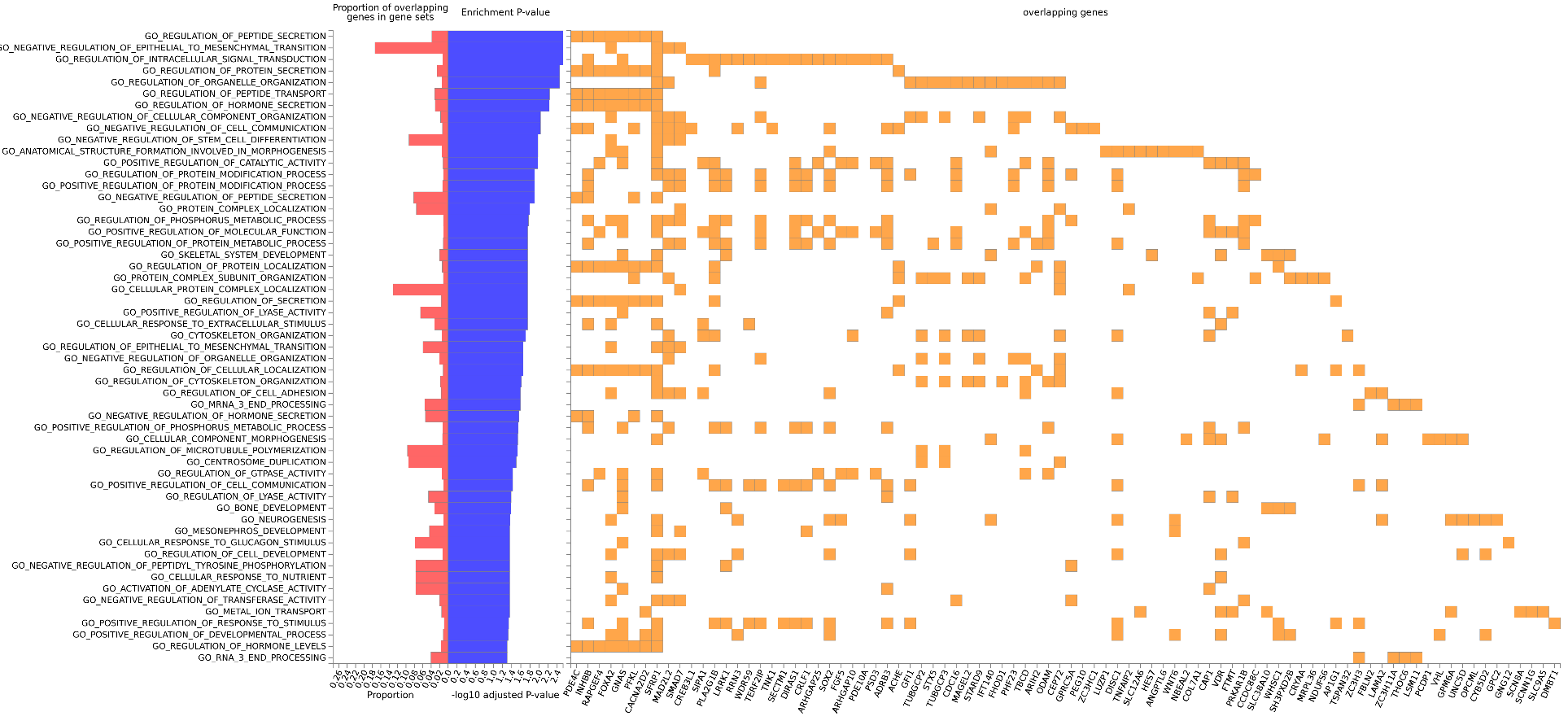
**

**Supplementary Figure 7. Biological processes identified for genes annotated to CpG sites within MRS.** Gene Ontology – biological processes, p-value enrichment for genes annotated to CpG sites, and overlapping genes in each gene-set (source: <https://fuma.ctglab.nl/>).

**
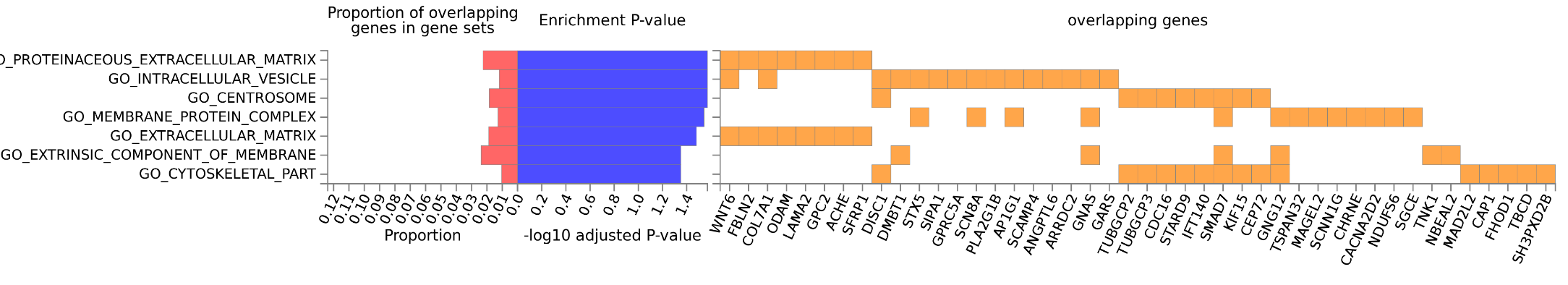
**

**Supplementary Figure 8. Cellular components identified for genes annotated to CpG sites within MRS.** Gene Ontology – cellular components, p-value enrichment for genes annotated to CpG sites, and overlapping genes in each gene-set (source: <https://fuma.ctglab.nl/>).

**MRS-ns**

**
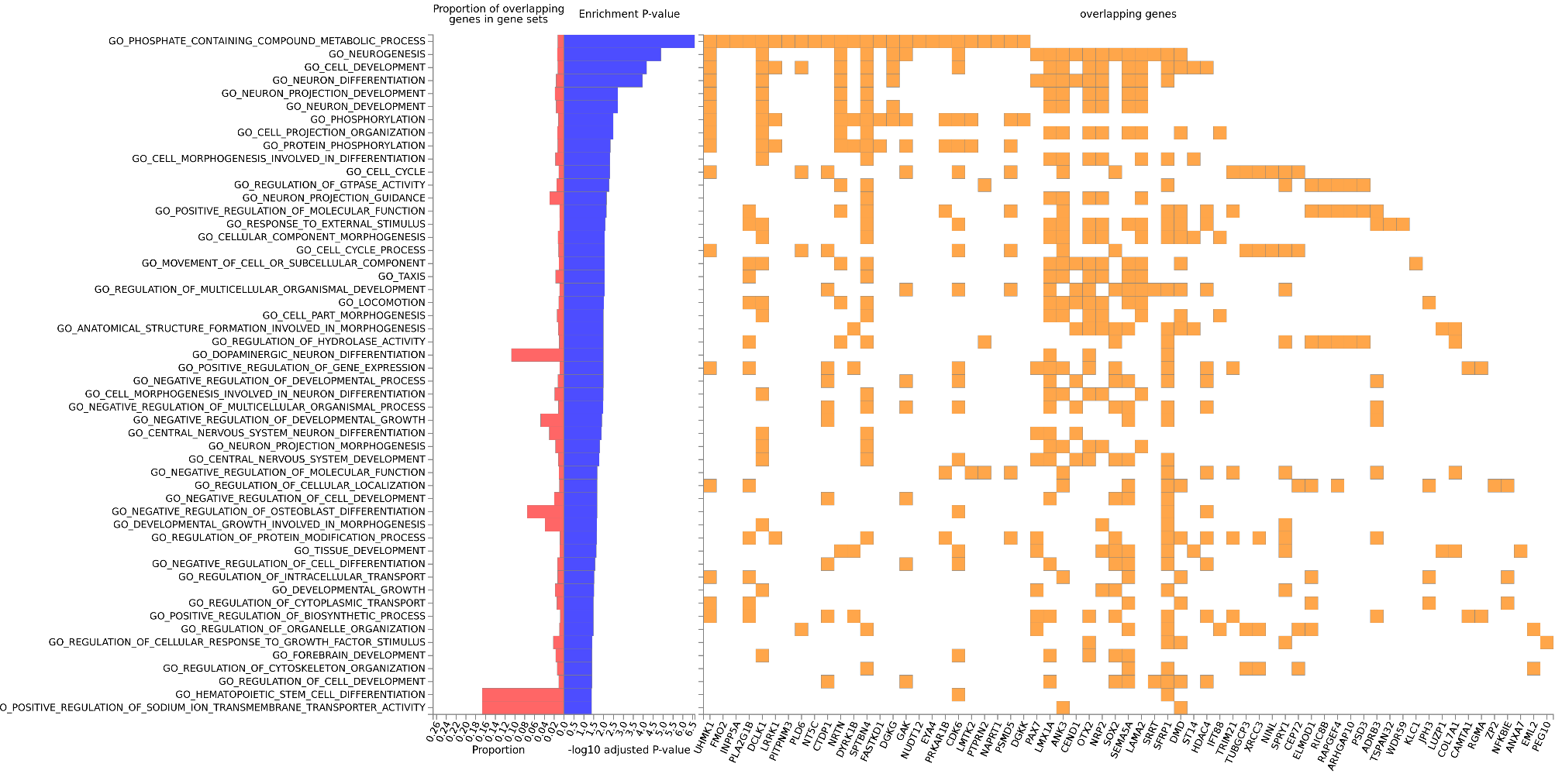
**

**Supplementary Figure 9. Biological processes identified for genes annotated to CpG sites within MRS-ns.** Gene Ontology – biological processes, p-value enrichment for genes annotated to CpG sites, and overlapping genes in each gene-set (source: <https://fuma.ctglab.nl/>).

**
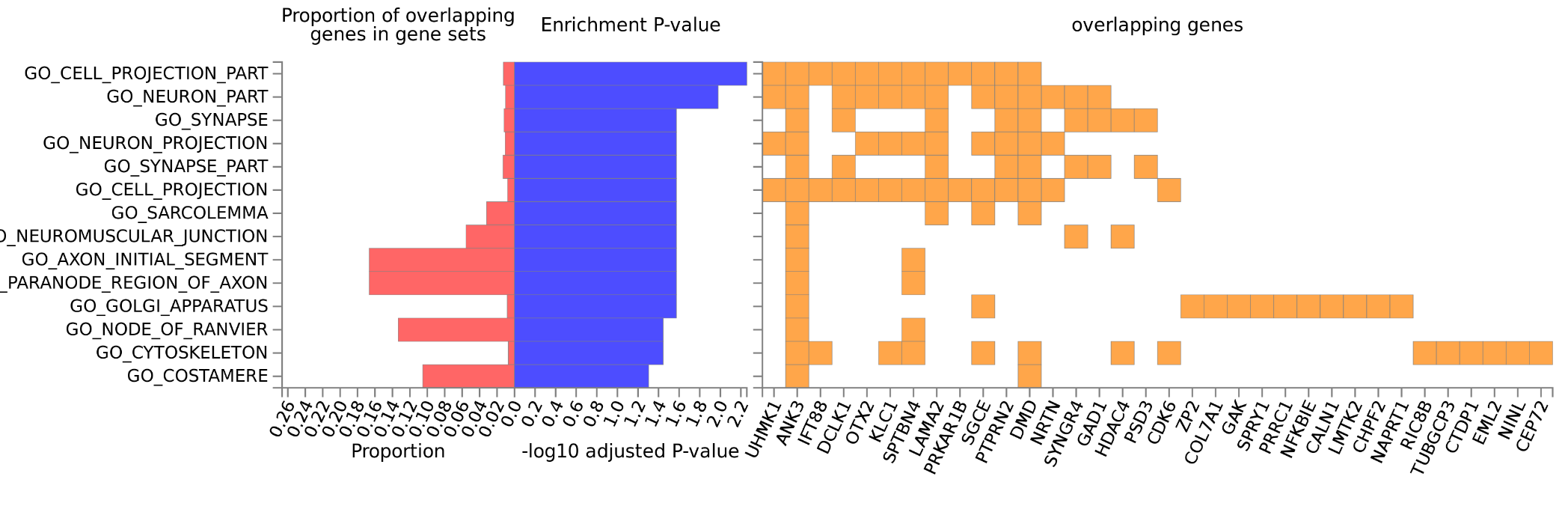
**

**Supplementary Figure 10. Cellular components identified for genes annotated to CpG sites within MRS-ns.** Gene Ontology – cellular components, p-value enrichment for genes annotated to CpG sites, and overlapping genes in each gene-set (source: <https://fuma.ctglab.nl/>).

**
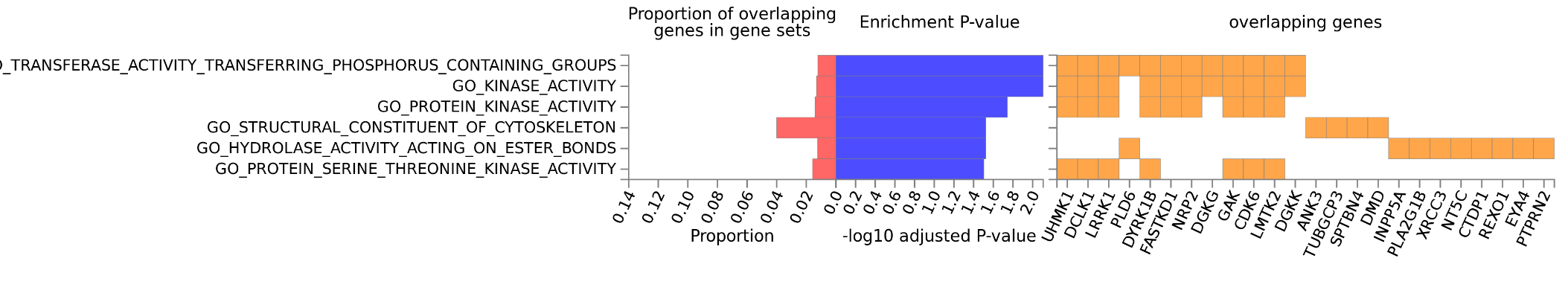
**

**Supplementary Figure 11. Molecular functions identified for genes annotated to CpG sites within MRS-ns.** Gene Ontology – molecular functions, p-value enrichment for genes annotated to CpG sites, and overlapping genes in each gene-set (source: <https://fuma.ctglab.nl/>).
